# CDC20 protects the heart from doxorubicin-induced cardiotoxicity by modulating CCDC69 degradation

**DOI:** 10.1186/s11658-025-00708-8

**Published:** 2025-03-05

**Authors:** Zhenyu Feng, Ningning Zhang, Liang Wang, Xumin Guan, Yunpeng Xie, Yun-long Xia

**Affiliations:** 1 Institute of Cardiovascular Diseases, The First Affiliated Hospital of Dalian Medical University, Lianhe Road 193, Dalian, Liaoning 116000 People’s Republic of China; 2 Department of Hematology, The First Affiliated Hospital of Dalian Medical University, Dalian, People’s Republic of China; 3 Department of Pharmacy, Liaoyang City Central Hospital, Liaoyang, People’s Republic of China

**Keywords:** Apoptosis, CDC20, CCDC69, Doxorubicin, Heart failure

## Abstract

**Aims:**

Doxorubicin (DOX) is a potent anticancer drug; however, it is associated with significant cardiotoxicity. CDC20 is an E3 ubiquitin ligase that plays a role in cell cycle progression and apoptosis in various types of cancers. The involvement of CDC20 in DOX-induced cardiotoxicity (DIC) is poorly understood. Hence, this study aimed to explore the potential role of CDC20 in the development of DIC and assess whether CDC20 influences the antitumor effects of DOX.

**Methods and results:**

H9C2 cells were treated with DOX, followed by transcriptomic analysis to identify differentially expressed genes. C57BL/6 mice were treated with DOX for 4 weeks after tail vein injection of CDC20 myocardial-specific knockout mice, AAV9-cTNT-(si) CDC20, or intraperitoneal injection of apcin. Cardiac function and pathological changes were evaluated by echocardiography and pathological staining, respectively. The influence of CDC20 on DOX-induced tumor inhibition was assessed in tumor-bearing mice. In vitro analysis involved treating cardiomyocytes with the Ad-CDC20 adenovirus and DOX, followed by proteomic and ubiquitination-related assays to identify potential downstream ubiquitinated CDC20 proteins. Additionally, we investigated the effect of CCDC69 on CDC20-mediated protection against DOX-induced apoptosis using CCDC69 shRNA. Transcriptome analysis revealed that DOX effectively suppressed the expression of CDC20. Cardiomyocyte-specific overexpression of CDC20 in a DOX-induced mouse model of myocardial injury effectively mitigated cardiomyocyte apoptosis, inflammation, fibrosis, and cell atrophy. Our mechanistic investigation revealed that CDC20 attenuates DOX-induced apoptosis by downregulating CCDC69 expression. Moreover, cardiomyocyte-specific overexpression of CDC20 had no effect on the therapeutic efficacy of DOX against tumors.

**Conclusion:**

Our findings indicate that CDC20 safeguards the heart against DOX-induced cardiotoxicity by modulating CCDC69 degradation without compromising the antitumor efficacy of DOX.

**Graphical Abstract:**

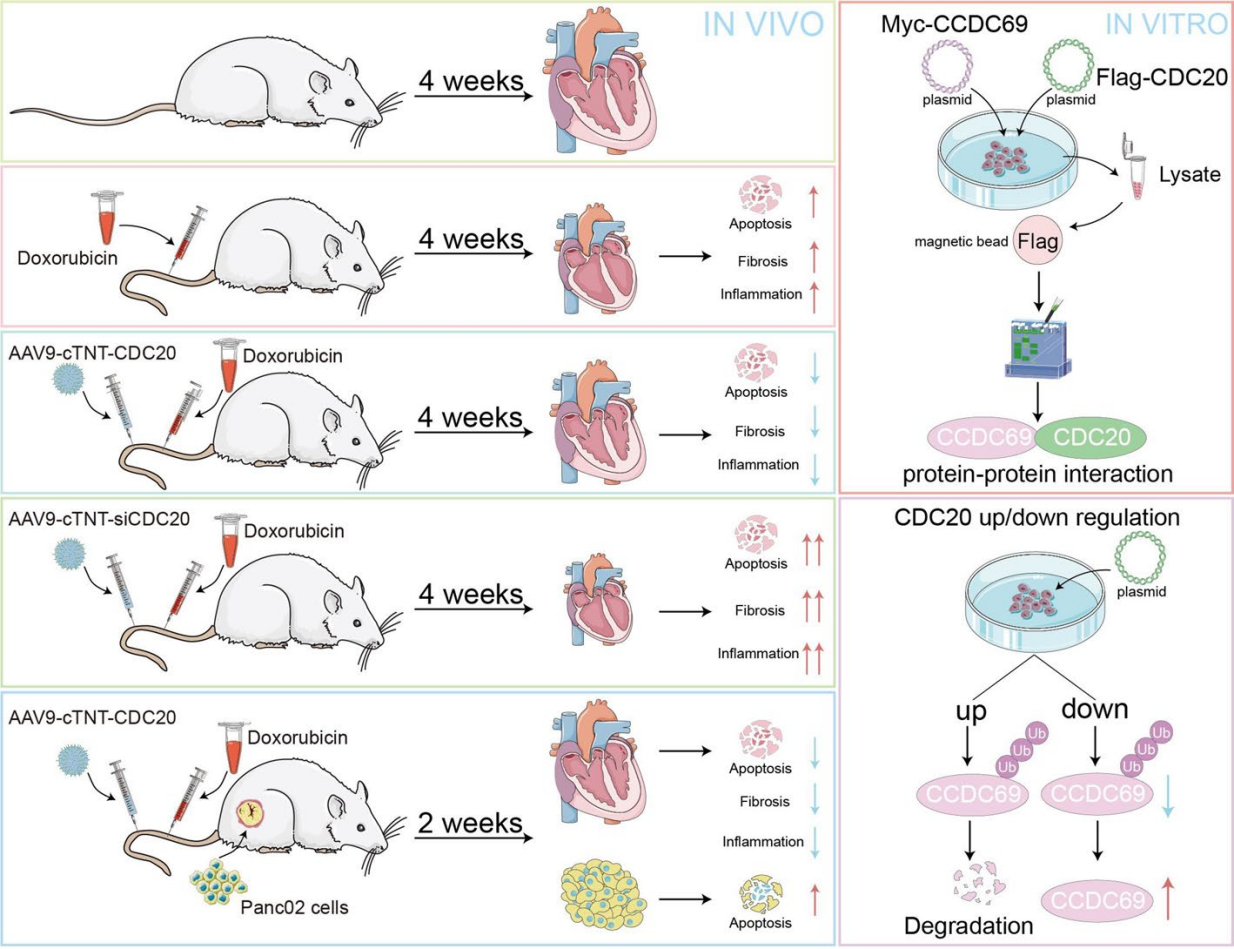

**Supplementary Information:**

The online version contains supplementary material available at 10.1186/s11658-025-00708-8.

## Introduction

Doxorubicin (DOX) is a broad-spectrum anthracycline antitumor drug used to treat various malignant tumors. However, studies have indicated that DOX has a high affinity for cardiomyocytes, leading to its accumulation in these cells. This results in severe cardiotoxicity and irreversible heart failure, which significantly hinder its clinical application [1, 2]. DOX-induced cardiotoxicity (DIC) typically occurs when the cumulative dose exceeds 500 mg/m^2^ [3]. However, recent research has revealed that, in 21% of patients, treatment-related cardiotoxicity can occur even at cumulative doses below 300 mg/m^2^ [4]. While the exact pathogenesis of DIC remains unclear, it is closely associated with cardiomyocyte apoptosis [5]. Therefore, drugs that may mitigate DOX-induced apoptosis in cardiac cells need to be identified.

Strategies to mitigate DIC involve the administration of liposomal DOX [6] or dexrazoxane [7]. However, their application is constrained by their potential for inducing secondary malignancies. Moreover, conventional medications for heart failure do not possess the ability to reverse or safeguard against anthracycline-induced cardiotoxicity. To identify and develop mitigation strategies for DIC, a mouse model of DOX-induced heart failure has been successfully developed, which has led to the identification of key indicators including cardiac weight loss, cardiomyocyte apoptosis, myocardial injury, and atrophy [8, 9].

Cell division cycle 20 (CDC20) plays a key role in the cell cycle and is crucial for cell proliferation. CDC20 activation triggers the activation of the anaphase-promoting complex [10]. Numerous studies have demonstrated that CDC20 is highly expressed in various malignant tumors, suggesting its involvement in tumor occurrence and progression [11]. Additionally, CDC20 protects diverse tumor cells from apoptosis. Our previous study revealed that CDC20 overexpression promotes Ang II-induced cardiomyocyte hypertrophy [12]. Therefore, we hypothesized that CDC20 confers protection against DOX-induced cardiomyocyte atrophy and apoptosis.

Coiled-coil domain containing 69 (CCDC69) is involved in the regulation of central spindle assembly, mainly by reducing microtubule stability, and the recruitment of essential components [13]. Survival analysis has demonstrated that high CCDC69 expression in patients with breast cancer correlates with improved overall survival (OS) and clinical prognosis [14]. Moreover, CCDC69 reduces cisplatin resistance in ovarian cancer by activating the p14ARF/MDM2/p53 signaling pathway [15].

This study explored the effects of increased CDC20 expression on DOX-induced cardiac injury in mice with the aim of contributing new information on potential molecular mechanisms and pathways that underpin DIC.

## Methods

### Animals

Male C57BL/6 mice aged 8–10 weeks were used in the experiments. The mice were randomly divided into multiple groups. CDC20-myh6 mice were obtained from VIEWSOLID (Beijing, China). CDC20-myh6 mice were intraperitoneally injected with tamoxifen (20 mg/kg/day, selleck, S1238) for 5 days, triggering gene recombination, followed by a further 2 weeks for tamoxifen clearance. The mice were intravenously injected with a cumulative dose of DOX at 20 mg/kg (5 mg/kg/week for 4 weeks, selleck, S1208). The mice were intraperitoneally injected with Apcin (selleck, S9605) at a dosage of 1.5 mg/kg/day. The Animal Care and Use Committee of Dalian Medical University approved all experimental procedures and protocols (AEE21079). All procedures were performed according to the guidelines from Directive 2010/63/EU of the European Parliament on the protection of animals used for scientific purposes or the NIH Guide for the Care and Use of Laboratory Animals.

### Bioinformatic analysis

Cells were treated with DOX (2 μM, 24 h) or vehicle. RNA was extracted using TRIzol (Invitrogen, 15596026CN). Sequencing analysis was conducted at Origingene (Shanghai Origingene Bio-pharm Technology Co., Ltd). Differentially expressed genes (DEGs) were considered with |log2| ≥ 1 and independent *t*-test *P* < 0.05.

Cells were treated with DOX (2 μM, 24 h) or DOX + Ad-CDC20. Protein analysis was performed at Origingene (Shanghai Origingene Bio-pharm Technology Co., Ltd). Differentially expressed proteins (DEPs) were considered with |log_2_| ≥ 0.58496 and independent *t*-test *P* < 0.05.

All data analysis (including Heatmap, volcano plot, GO, KEGG, and Venn) and visualization of differentially expressed genes (DESeq2) were performed using Xiantao (www.xiantao.love/).

### Echocardiography

Echocardiography was performed with 2% isoflurane and maintained in anesthesia by 1% isoflurane. Echocardiography was utilized to assess cardiac function in mice under superficial anesthesia using a Vevo 2100 high-resolution imaging system (Visualsonic, LTD.). Subsequently, the left ventricular ejection fraction (EF%) and fractional shortening (FS%) were calculated. After echocardiography, the mice were euthanized by heart resection under tribromoethanol anesthesia.

### Histopathology

Heart tissues were fixed in 4% formalin for 24 h at the conclusion of the experiment and subsequently embedded in paraffin. Subsequently, the 5-μm tissue sections were sliced and stained with hematoxylin–eosin (H&E) (H&E, Solarbio, G1120) and Masson (Solarbio, G1340). Lastly, the histopathologic slides were observed and evaluated using a light microscope (Olympus, BX53).

### Immunohistochemistry

Tissue sections were subjected to immunohistochemistry by treating them with 3% H_2_O_2_ to inhibit endogenous peroxidase activity. Subsequently, the sections were incubated with primary antibodies (Table 1) overnight at 4 °C. Following this, the sections were incubated with the appropriate secondary antibody and diaminobenzidine (DAB, ZSGB-BIO, ZLI-9018), and counterstained with hematoxylin.

**Table 1:** Information on antibodies

Name	Vendor	Cat. no.
Bax	ABclonal	A19684
Bcl-2	ABclonal	A0208
CCDC69	NOVUS	NBP1-77145
CCDC69	Invitrogen	PA5-65748
CDC20	Abcam	ab183479
Flag	Proteintech	20543-1-AP
MYC	Proteintech	60003-2-lg
Ub	Proteintech	10201-2-AP
HA	Proteintech	51064-2-AP
PCNA	ABclonal	A0264
CD68	Arigo	ARG10514
α-SMA	Arigo	ARG66381
Collagen III	Proteintech	22734-1-AP
β-actin	Affinity	T0022
PARP1	ABclonal	A0942
Caspase-3	ABclonal	A11319

### Immunofluorescence

Cultured cells or heart paraffin sections were incubated overnight at 4 °C with specific primary antibodies (Table 1). Subsequently, a goat anti-rabbit IgG (H + L) highly cross-adsorbed secondary antibody, Alexa Fluor™ Plus 488 (Invitrogen, A32731) or goat anti-mouse IgG (H + L) highly cross-adsorbed secondary antibody, Alexa Fluor™ Plus 555 (Invitrogen, A32727) was used as the secondary antibody and incubated for 1 h at room temperature. Finally, the sections were observed with fluorescence microscopy (Olympus, BX53).

### Wheat germ agglutinin (WGA)

The tissue sections were dewaxed and rehydrated, followed by a 60-min incubation with WGA staining (2 ng/ml, VECTOR, RL-1002). The surface area of cardiomyocytes was measured using ImageJ software.

### Western blot analysis

Proteins (30 μg) were loaded and separated using 10% or 12.5% sodium dodecyl sulfate–polyacrylamide gel electrophoresis (SDS-PAGE), and subsequently transferred onto polyvinylidene fluoride (PVDF) membranes. The membranes were blocked with 5% skim milk for 1 h at room temperature, followed by overnight incubation with specific primary antibodies (Table 1) at 4 °C. On the following day, the membranes were incubated with HRP-coupled secondary antibodies or IRDye 800CW goat anti-rabbit antibody (LI-COR,926-32211) or IRDye 800CW goat anti-mouse antibody (LI-COR,926-32210), and protein bands were visualized using an electrochemiluminescence reagent or the Odyssey DLx infrared imaging system.

### TDT-mediated dUTP nick end-labeling (TUNEL) staining

Cardiac apoptosis in myocardial tissue was assessed using a TUNEL assay kit (C1091, Beyotime) following the manufacturer’s protocol. Subsequently, cardiac apoptosis was observed and evaluated using a light microscope (Olympus, BX53).

### Cell culture and treatment

H9C2 (CL-0089), AC16 (CL-0790), B16-F10 (CL-0319), Panc02 (CL-0736), and 293 T (CL-0005) cells were sourced from Procell Life Science & Technology Co., Ltd. H9C2, AC16, and 293 T cells were cultured in high-glucose DMEM, while Panc02 and B16-F10 cells were cultured in RPMI1640 supplemented with 10% fetal bovine serum (FBS) and 1% antibiotics (1% penicillin and streptomycin) at 37 °C in 5% CO_2_.

Rat neonatal cardiac myocyte were isolated from the hearts of 24-h-old SD rats, all of which was performed under a sterile hood. After the hearts were removed, the hearts were cut to about 1 mm^3^ with scissors, digested with trypsin–EDTA solution at 37 °C, and collected. Then, the cells were resuspended using 10% FBS/DMEM/F12 medium and incubated on 100-mm culture dishes for 90 min at 37 °C with 5% CO_2_ differential adhesion. Cardiomyocytes were resuspended, collected, and transferred to either 6-well plates or 24-well plates. Cardiomyocytes were cultured in DMEM/F12 containing 10% FBS.

### Adenoviruses and cell infection

The H9C2 cells were pretreated with either Ad-CDC20 or GFP adenovirus for 24 h after reaching 70–80% confluency, followed by stimulation with DOX (2 μM) for another 24 h. Ad-CDC20 and GFP adenoviruses were obtained from Hanbio Technology (Shanghai, China) Co., Ltd.

### Transfection

The AC16 cells and 293 T cells were transfected with Flag-CDC20 (1–499, 1–167, 168–477, and 168–499, pcDNA3.1-EF1a-mcs-3flag-CMV-EGFP), Myc-CCDC69 (pcDNA3.1-EF1a-MCS-CMV-EGFP), HA-UB (pcDNA3.1), shCDC20 (PCDNA3.1-U6-MCS-CMV-ZsGreen-T2A-PURO, sequence: GAT CCG TGG TGG TAA TGA TAA CTT GGT CTC GAG ACC AAG TTA TCA TTA CCA CCA TTT TTT G), and shControl (sequence: GAT CCG TTC TCC GAA CGT GTC ACG TAA TTC AAG AGA TTA CGT GAC ACG TTC GGA GAA TTT TTT C. PCDNA3.1-U6-MCS-CMV-ZsGreen-T2A-PURO) plasmids using LipoFiter 3.0™ reagent, following the manufacturer’s protocol, when they reached 70–80% confluency. The plasmids and LipoFiter 3.0™ reagent were obtained from Hanbio Technology (Shanghai, China) Co., Ltd.

### RNA isolation and real-time PCR

Total RNA was extracted from cardiac tissues or cells using the TRIzol (Invitrogen, 15596026CN) reagent. Subsequently, cDNA synthesis was performed using a cDNA synthesis kit (YEASEN, 11141ES), and real-time quantitative PCR was conducted using SYBR Green Master Mix (YEASEN, 11184ES). The primer sequences are listed in Table 2.

**Table 2:** List of primers used for real-time quantitative PCR

Gene	Forward primer (5′–3′)	Reverse primer (5′–3′)
Rat CDC20	TCAAGGTGCTGTCAAGGCTG	GAGCAGACGTTCCAAATGCG
Rat β-actin	GAATCAGATAGAGCCCGGCG	CCAGGCAGTGAAAGTCTTCCT
Mouse CDC20	TTCGTGTTCGAGAGCGATTTG	ACCTTGGAACTAGATTTGCCAG
Mouse CCDC69	TCCCGCAGACATCGAATGC	TCCTGTGGTGAGACATGACTT
Mouse β-actin	GTGACGTTGACATCCGTAAAGA	GCCGGACTCATCGTACTCC
Human CDC20	GCACAGTTCGCGTTCGAGA	CTGGATTTGCCAGGAGTTCGG
Human CCDC69	GTTCTACCCAGCAGGAGACC	GGCTGGGGCTCCCATAATC
Human β-actin	GAGAAAATCTGGCACCACACC	GGATAGCACAGCCTGGATAGCAA

### Immunoprecipitation assay

To confirm the interaction between CDC20 and CCDC69 using co-immunoprecipitation (co-IP), 293 T cells were transfected with plasmids overexpressing Flag-CDC20 and MYC-CCDC69. The cell lysates obtained from the immunoprecipitation were incubated overnight at 4 °C with anti-Flag and anti-MYC antibodies (Table 1). Elution was performed by adding 1 × SDS buffer for 5 min, followed by centrifugation. The samples were then subjected to Western blot analysis.

### In vivo antitumor efficacy

C57BL/6 J (8 weeks, ~22 g, male, strong physique, strong limbs, smooth and shiny coat without trauma) received rAAV9-cTNT-CDC20 and rAAV9-cTNT-control 2 weeks in advance through a tail vein. Panc02 and B16-F10 cells at logarithmic growth stage were digested, centrifuged, and counted, and cell suspension was prepared by mixing PBS and Matrigel (ABW, 0827245) 1:1. Mice were grabbed, and the hair on the right back including the thigh of the mice was shaved with a small animal shaver, and the skin was disinfected with iodine. At the base of the right thigh of the mouse, a sterile syringe needle was inserted under the skin of the mouse in a direction parallel to the mouse skin. When the needle is slightly lifted, it can be seen that the epidermis of the mouse is obviously lifted, which proves that it has penetrated into the subcutaneous layer instead of the muscle layer, and the position is correct. Panc02 and B16-F10 tumor-bearing mice were established subcutaneously inoculation of Panc02 and B16-F10 cells into the right flank of mice (1 × 10^6^ cells/200 μL media and 8 × 10^5^ cells/200 μL media). The speed of injection should not be too fast. The needle was slowly withdrawn, and the mice were returned to the cage for rearing. Seven days after implantation, mice were randomly divided into four groups: control group, Panc02 or B16-F10 tumor group, Panc02 or B16-F10 tumor + DOX group, and Panc02 or B16-F10 tumor + DOX + rAAV9-CDC20 group. Then, after the model was successful, we observe the physiological state of the mice every day, such as weight, coat color, diet, etc. Cardiac ultrasounds are also performed on the mice weekly, while it is unethical to observe the size of the tumor every other day. Mice received DOX through the tail vein, and the cumulative dose of DOX was 20 mg/kg or 10 mg/kg. Tumor volumes were calculated as *V* = length × width^2^/2 (in mm). Tumor volumes and body weights of mice were recorded every day.

### Cycloheximide and MG132 assays

NRCMs were infected with Ad-CDC20 or GFP adenovirus for 24 h. Cycloheximide (CHX, 100 μg/ml) was added at 0, 3, 6, 9 h, respectively, and MG132 (10 μM) was added 6 h in advance. The cell lysate was collected, and the level of ccdc69 protein was detected by western blot analysis using anti-ccdc69 Ab.

### Caspase 3 activity assay

The cells and tissues of each group were collected into the centrifuge tube, and the lysis buffer was added according to the number of cells (Solarbio, BC3830), and then centrifuged at 4 °C, 15,000*g*, for 15 min. The protein concentration was determined by Bradford method, and then diluted to 1 mg/ml. Ac-DEVD-pNA substrate and reaction buffer were added according to the instructions. Incubation was performed at 37 °C for 1 h, and the absorbance at 405 nm was measured with a preheated enzyme marker. The standard equation is derived from the concentration of the standard tube (*x*, μmol/L) and ∆*A* standard (*y*, minus the absorbance of the standard tube with a concentration of 0). Substituting the measurement of ∆*A* into the standard equation gives *x* (μmol/L).

### Statistical analysis

The statistical analysis results are presented as mean ± standard deviation (SD). Group comparisons were performed using Student’s *t*-test or one-way ANOVA (corrected for multiple comparisons using Bonferroni’s method in GraphPad Prism 9.0) or two-way ANOVA. A significance level of *P* < 0.05 was used to determine statistical significance.

## Results

### Doxorubicin-induced cardiac injury and apoptosis

**Fig. 1:**
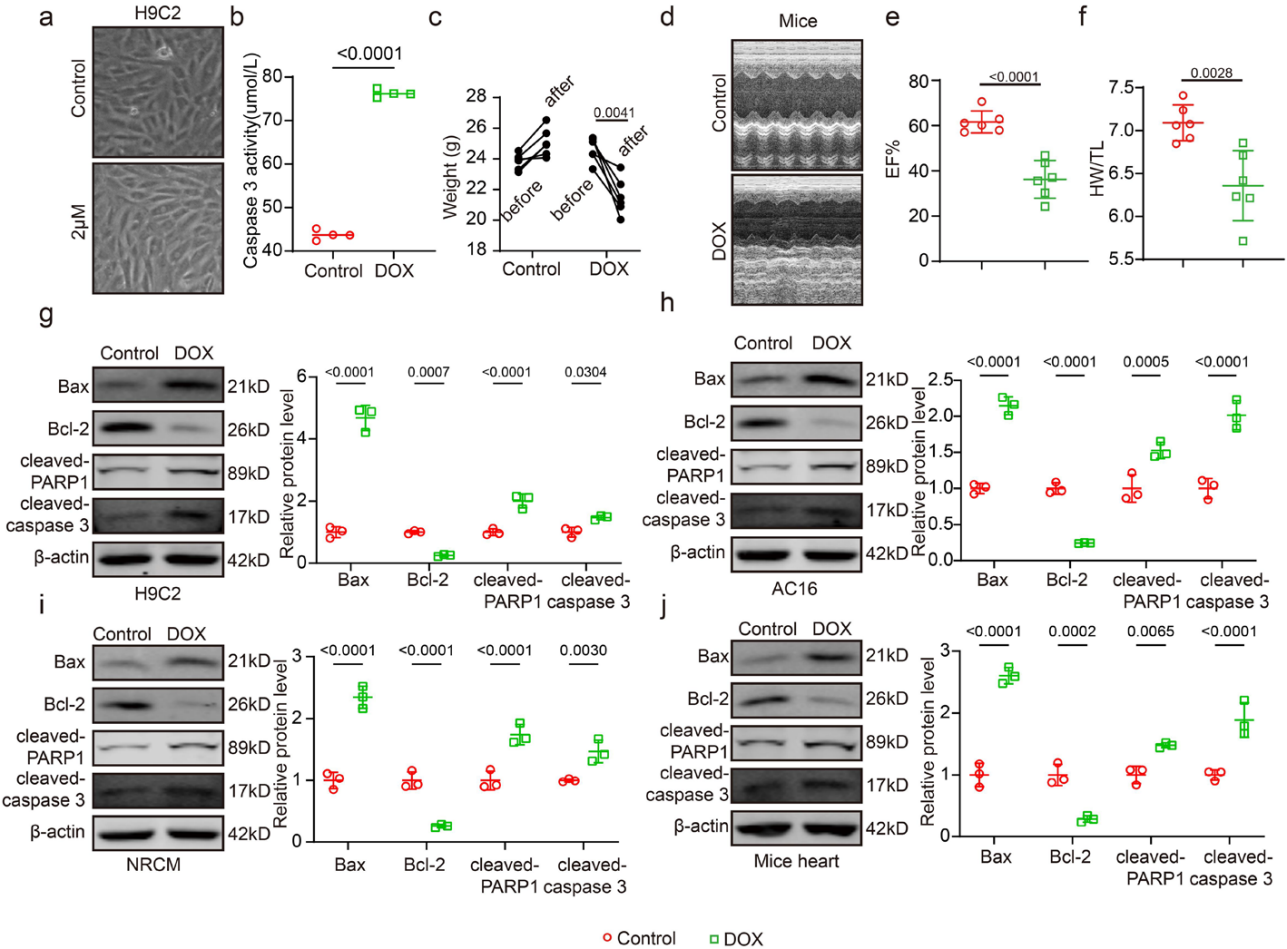
Doxorubicin-induced cardiac injury and apoptosis. **a** The cellular morphological changes in control group and DOX (2 μM) group were observed under a microscope (*n* = 3, bar = 50 μm); **b** Caspase 3 activity in control group and DOX (2 μM) (*n* = 4); **c** Weight change of mice in control group and DOX (20 mg/kg) (*n* = 6); **d** Representative image of cardiac function in control group and DOX (20 mg/kg) (*n* = 4); **e** EF% statistics of mice in control group and DOX (20 mg/kg) (*n* = 4); **f** HW/TL statistics of mice in control group and DOX (20 mg/kg) (*n* = 4); **g**–**j** Measured expression levels of Bax, Bcl-2, and β-actin in H9C2, AC16, NRCM at 2 μM concentration of DOX, and mouse hearts at 20 mg/kg concentration of DOX (*n* = 3). EF: ejection factor, HW/TL: heart weight/tibia length. Data presented as mean ± SD. Statistical analysis performed with Student’s *t* test

Initially, we examined the potential damage to cardiomyocytes induced by DOX in vitro. After 24 h of stimulating H9C2 rat embryonic cardiomyocytes with DOX (2 μM), we observed vacuolization and a reduction in cell count (Fig. 1a). Furthermore, caspase 3 activity increased following DOX treatment (2 μM) (Fig. 1b). Subsequently, we assessed the potential adverse effects of DOX on the mouse hearts. After 4 weeks of DOX treatment (20 mg/kg), mice exhibited increased body weight in the control group, whereas those experienced a significant decrease in the DOX-treated group (after versus before) (Fig. 1c). Ultrasound detection revealed a significant decrease in the ejection fraction (EF) of DOX-treated mice (Fig. 1d, e). In addition, the heart weight/tibia length (HW/TL) ratio of mice significantly decreased after DOX treatment (Fig. 1f). Subsequently, we examined the expression of the apoptosis-related proteins, BAX, and Bcl-2, in three types of cardiomyocytes lines (H9C2, AC16, and neonatal rat cardiomyocytes [NRCM]) and mouse heart tissues. BAX expression was significantly upregulated, and Bcl-2 expression significantly downregulated in H9C2 (Fig. 1g), AC16 (Fig. 1h), NRCM (Fig. 1i), and mouse heart tissue (Fig. 1j) after DOX treated (2 μM). These findings were consistent with those of previous studies.

### CDC20 suppresses DOX-induced myocardial cell damage

**Fig. 2:**
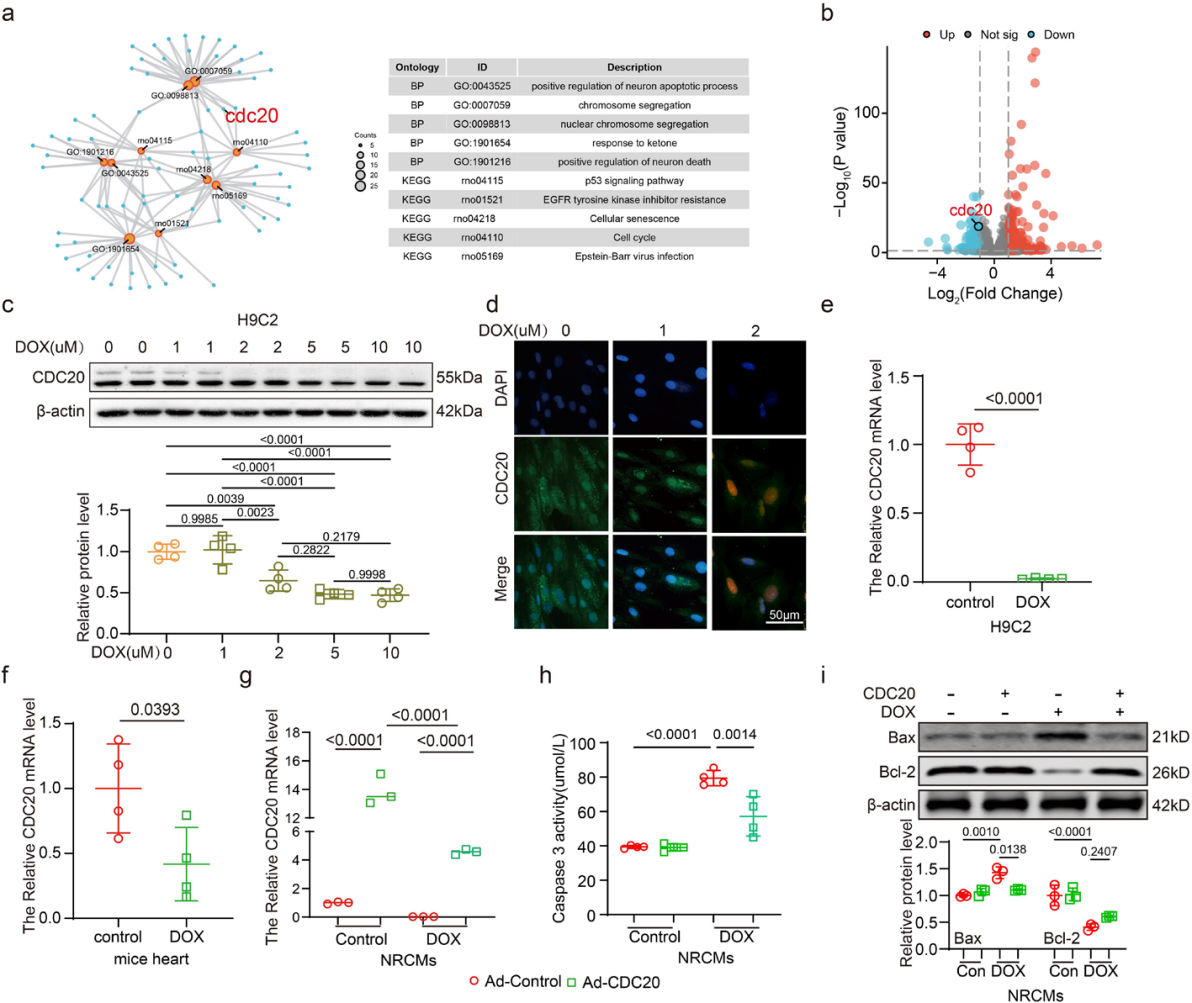
Intervention of CDC20 in DOX-induced myocardial cell damage. **a** Results of GO and KEGG analyses on the differentially expressed genes between the control group and DOX treatment; **b** Volcano plot illustrating gene expression and CDC20 expression; **c** Expression of CDC20 and β-actin using western blotting (*n* = 4); **d** Immunofluorescence staining to detect CDC20 expression in control group and DOX (1 and 2 μM) of H9C2 cells (*n* = 3, bar = 50 μm); **e** Quantified expression of CDC20 in H9C2 cells using qPCR in control group and DOX (2 μM) (*n* = 4); **f** Expression of CDC20 in the hearts of mice using qPCR (*n* = 4); **g** Expression of CDC20 in NRCMs cells using qPCR (*n* = 3); **h** Caspase-3 activity in each experimental group (*n* = 4); **i** Expression of Bax, Bcl-2, and β-actin using western blotting (*n* = 3). DOX: doxorubicin, GO: Gene Ontology, KEGG: Kyoto Encyclopedia of Genes and Genomes, qPCR: quantitative polymerase chain reaction. Data presented as mean ± SD. Statistical analysis performed with one-way ANOVA or two-way ANOVA

We conducted a transcriptomics analysis of H9C2 cells treated with 2 μM DOX to analyze the downstream genes regulated by DOX. In total, 12,065 genes were identified. Based on thresholds of |log2(fold change [FC])|> 1 and *p* < 0.05, we found 444 differentially expressed genes, including 206 upregulated (positive log2(FC)) and 238 downregulated (negative log2(FC)) gene. Gene Ontology (GO) and Kyoto Encyclopedia of Genes and Genomes (KEGG) enrichment analyses revealed that CDC20 was significantly downregulated by DOX (Fig. 2a, b). We treated H9C2 cells with different concentrations of DOX (0, 1, 2, 5, 10 μM) to detect the expression of CDC20. The results showed that the expression of CDC20 was significantly reduced at the DOX concentration of 2 μM compared with the control group, but CDC20 was not significantly different at the DOX concentration of 5 μM and 10 μM compared with the DOX concentration of 2 μM. Therefore, in the next experiment, we always treated H9C2 cells with DOX at 2 μM concentration (Fig. 2c). Immunofluorescence analysis corroborated that CDC20 expression was significantly reduced in H9C2 cells after DOX stimulation (2 μM) (Fig. 2d). Furthermore, quantitative (q)PCR results confirmed the inhibition of CDC20 expression following DOX stimulation both in vivo and in vitro (Fig. 2e,f), as did western blotting analysis of mouse hearts (Supplementary Fig. S1a), AC16 cells (Supplementary Fig. S1b), and NRCMs (Supplementary Fig. S1c).

Next, NRCMs were infected with a recombinant adenovirus overexpressing CDC20 (Ad-CDC20) and stimulated with DOX. Infection with Ad-CDC20 increased the CDC20 expression level as compared with that in the control group. Furthermore, CDC20 expression was significantly higher in the Ad-CDC20 + DOX group than in the DOX group (Fig. 2g). While DOX stimulation enhanced caspase 3 activity, caspase 3 activity was reduced in the DOX + Ad-CDC20 group compared with that in the DOX group (Fig. 2h). Further, CDC20 overexpression inhibited the increase in Bax expression and downregulation of Bcl-2 expression induced by DOX (Fig. 2i). Finally, CDC20 inhibition using Ad-siCDC20, encoding an siRNA targeting CDC20, or the CDC20 inhibitor apcin exacerbated DOX-induced cardiomyocyte apoptosis (Supplementary Figs. S2 and S3).

### Overexpression of CDC20 attenuates DOX-induced cardiac dysfunction, fibrosis, and apoptosis

**Fig. 3:**
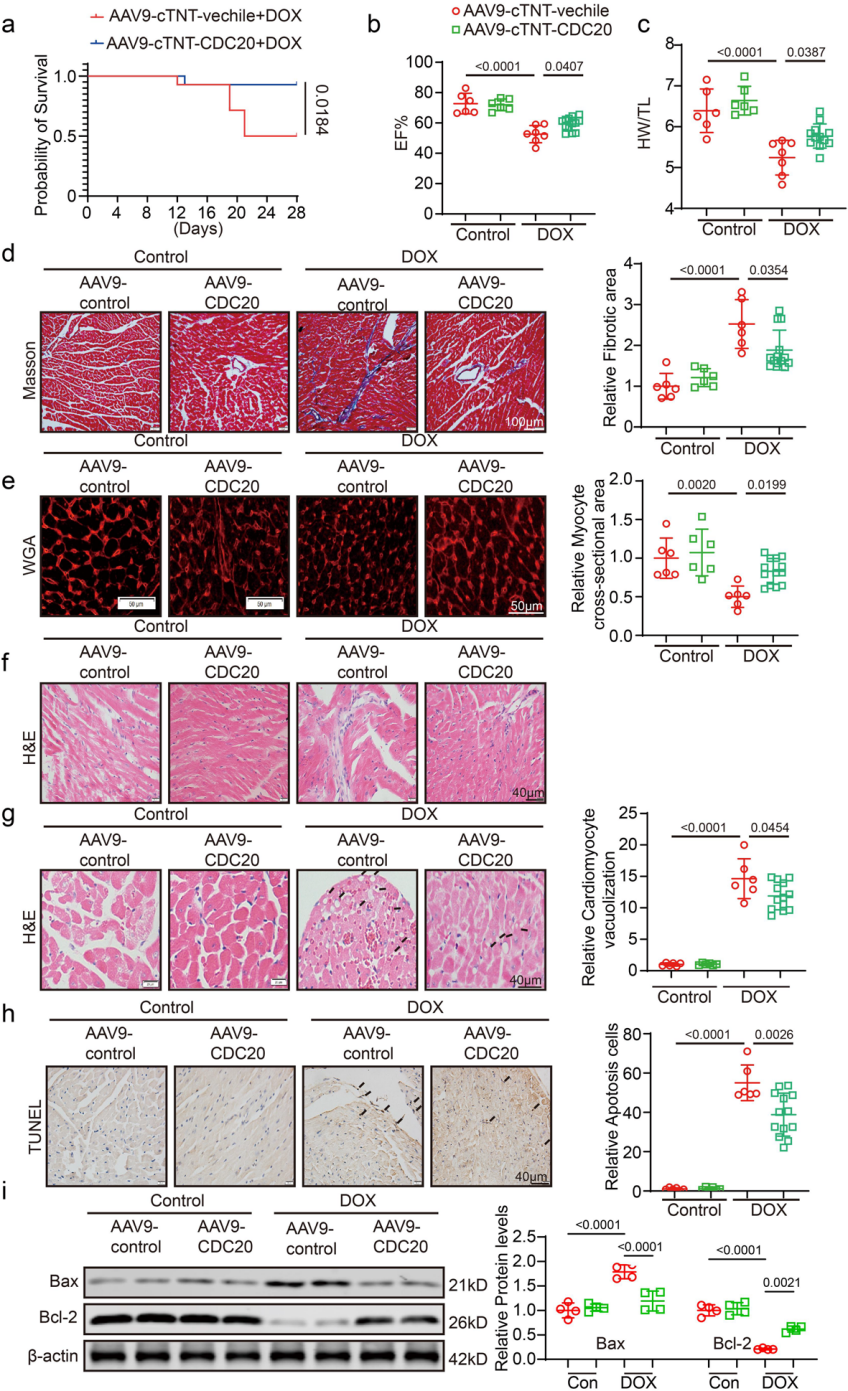
Overexpression of CDC20 attenuates DOX-induced cardiac dysfunction, fibrosis, and apoptosis. **a** Survival curve of mice in each group: **b** EF% statistics of mice in each group (*n* = 6–13); **c** HW/TL statistics of mice in each group (*n* = 6–13); **d** Representative image of Masson staining in each group (*n* = 6–13, bar = 100 μm); **e** Representative image and statistics of WGA staining in each group (*n* = 6–13, bar = 50 μm); **f** HE staining image in each group (*n* = 6–13, bar = 40 μm); **g** Representative image and statistical data of vacuolization in each group (*n* = 6–13, bar = 40 μm); **h** Representative image and statistical data of TUNEL staining in group (*n* = 6–13, bar = 40 μm); **i** Detected expression of Bax, Bcl-2, and β-actin using western blotting (*n* = 4). DOX: doxorubicin, EF: ejection factor, H&E: Hematoxylin and eosin, HW/TL: heart weight/tibia length, TUNEL: terminal deoxynucleotidyl transferase dUTP nick end labeling, WGA: wheat germ agglutinin. Data presented as mean ± SD. Statistical analysis performed with two-way ANOVA

To investigate the role of CDC20 in mouse cardiomyocytes, CDC20 was overexpressed in mice via intravenous tail injection of an adeno-associated virus (AAV9) encoding the protein. The efficacy of AAV9-CDC20 infection in mouse hearts was assessed using PCR. CDC20 expression in mouse hearts was indeed significantly increased following AAV9-CDC20 infection (Supplementary Fig. S4a). In the DOX-induced cardiac injury mouse model (Fig. 3a), mortality was significantly increased (mortality rate close to 50%) compared with that in the control group. However, myocardial-specific CDC20 overexpression notably improved survival. DOX administration led to a decrease in the EF compared with that in the control group (AAV9-cTNT-vehicle), whereas CDC20 overexpression significantly increased the EF% compared with that in the DOX group (Fig. 3b). Similarly, DOX treatment decreased the HW/TL compared with AAV9-cTNT-vehicle treatment, while CDC20 overexpression significantly increased this ratio (Fig. 3c).

Masson’s trichrome staining was used to assess the fibrotic status of mouse hearts. DOX administration induced cardiac fibrosis, whereas CDC20 overexpression significantly reduced the level of cardiac fibrosis compared with that in the DOX group (Fig. 3d). Cardiomyocyte size was measured using wheat germ agglutinin (WGA) staining. DOX administration led to cardiomyocyte atrophy, whereas cardiomyocytes size was restored by CDC20 overexpression (Fig. 3e). Hematoxylin and eosin (H&E) staining revealed that cardiac inflammatory aggregates and vacuolation were increased after DOX administration, but suppressed by CDC20 overexpression (Fig. 3f, g). The degree of cardiomyocyte apoptosis was assessed using terminal deoxynucleotidyl transferase dUTP nick end labeling (TUNEL) staining and western blotting. DOX administration increased the degree of cardiac apoptosis, while the degree of cardiac apoptosis decreased after CDC20 overexpression (Fig. 3h, i).

### Inhibition of CDC20 aggravates DOX‑induced cardiac dysfunction, fibrosis, and apoptosis

**Fig. 4:**
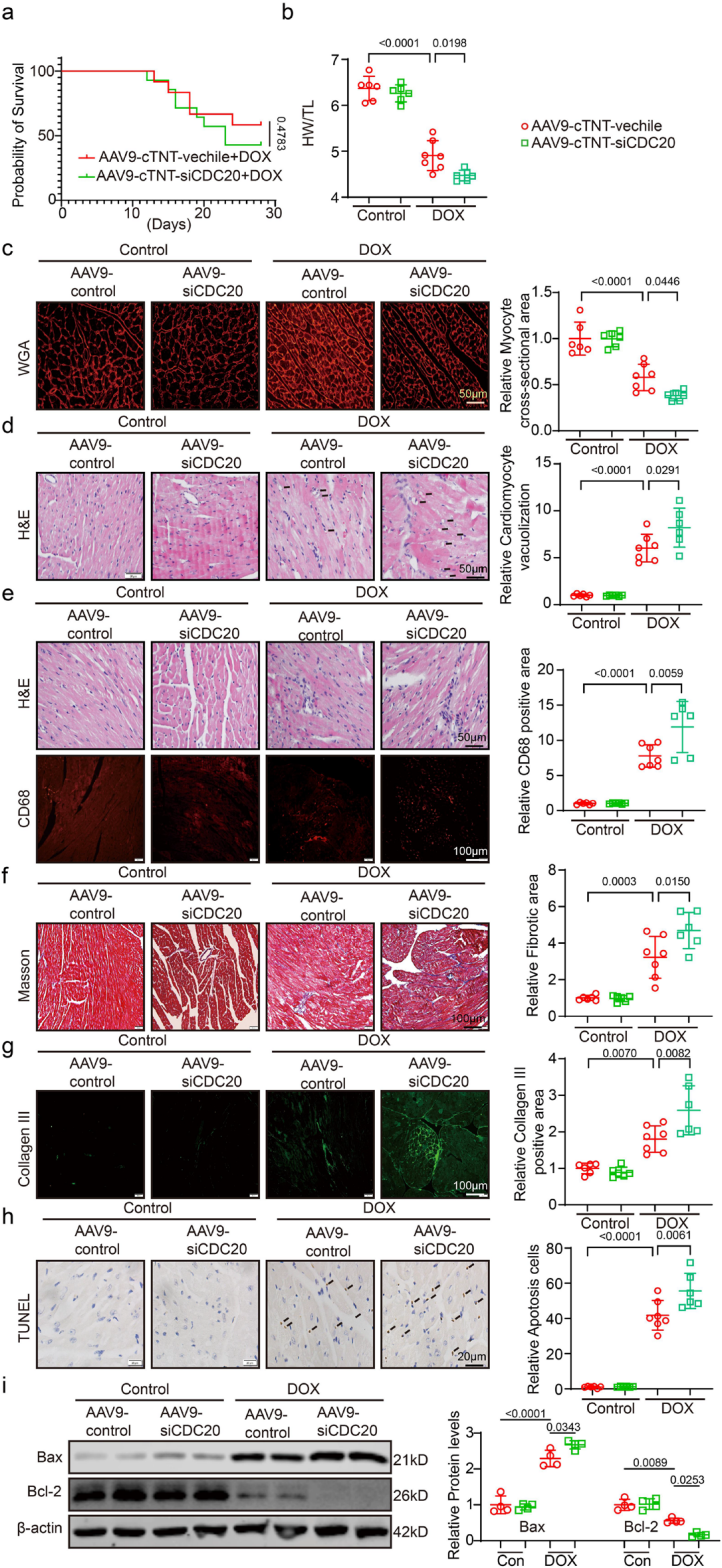
Inhibition of CDC20 aggravates DOX‑induced cardiac dysfunction, fibrosis, and apoptosis. **a** Survival curves in each group; **b** HW/TL statistics of mice in each group (*n* = 6–7); **c** Representative image and statistical data of WGA staining in each group (*n* = 6, bar = 50 μm); **d** Representative images and statistics of vacuolization in each group (*n* = 6–7, bar = 50 μm); **e** Representative image of HE staining (bar = 50 μm), CD68 staining (bar = 50 μm), and statistical data in each group (*n* = 6–7); **f** Representative images and statistical data of Masson staining in each group (*n* = 6–7, bar = 100 μm); **g** Representative images and statistical data of Collagen III staining in each group (*n* = 6–7, bar = 100 μm); **h** Representative images and statistical data of TUNEL staining in each group (*n* = 6–7, bar = 20 μm); **i** Detection of Bax, Bcl-2, and β-actin expression through western blotting (*n* = 4). DOX: doxorubicin, H&E: Hematoxylin and eosin, HW/TL: heart weight/tibia length, TUNEL: terminal deoxynucleotidyl transferase dUTP nick end labeling, WGA: wheat germ agglutinin. Data presented as mean ± SD. Statistical analysis performed with two-way ANOVA

The above findings demonstrated that CDC20 overexpression protected against DOX-induced myocardial injury. Therefore, we next investigated whether the inhibition of CDC20 expression would exacerbate DOX-induced myocardial injury. CDC20 expression in cardiomyocytes of DIC model mice was suppressed by injecting AAV9-cTNT-siCDC20 via the tail vein. Data demonstrating the silencing efficacy are presented in Supplementary Fig. S4b. DOX significantly increased mouse mortality by nearly 40% compared with the control. Myocardial-specific CDC20 silencing further increased the death rate, albeit not significantly (Fig. 4a). Similarly, DOX treatment resulted in a decrease in HW/TL. After CDC20 inhibition, the HW/TL value further decreased (Fig. 4b). WGA staining analysis revealed that DOX administration induced cardiomyocyte shrinkage, which was further aggravated by CDC20 knockdown (Fig. 4c). DOX increased the extent of cardiac vacuolation as well as inflammatory cell aggregation compared with the control as indicated by H&E staining, and CDC20 knockdown further promoted these phenomena (Fig. 4d). CD68 expression in the mouse hearts was significantly enhanced after DOX treatment as indicated by immunofluorescence staining, and even more so after CDC20 knockdown (Fig. 4e). DOX administration induced cardiac fibrosis, which was further increased after CDC20 knockdown, as indicated by Masson’s trichrome staining (Fig. 4f). Further, DOX administration significantly increased collagen III expression compared with the control as revealed by fluorescence analysis, and collagen III expression was further enhanced after CDC20 knockdown (Fig. 4g). Using TUNEL staining and Western Blot, we found that DOX administration significantly increased the extent of cardiac apoptosis, and CDC20 knockdown exacerbated this effect (Fig. 4h, i). These findings based on overexpression and interference experiments demonstrated the significant role of CDC20 in DOX-induced myocardial injury using overexpression and interference experiments. For further validation, we used cardiac-specific CDC20 knockout mice and the CDC20-specific inhibitor, apcin, for further validation. Both CDC20 knockout and apcin treatment exacerbated DOX-induced cardiac dysfunction, fibrosis, and apoptosis (Supplementary Figs. S6 and S7).

### CDC20 is capable of ubiquitinating CCDC69 in cardiomyocytes

Proteomic analysis was conducted on the control, DOX, and DOX + CDC20 groups. The numbers of differentially expressed proteins were visualized in Venn diagrams (Supplementary Fig. S8a). Specifically, 209 proteins were downregulated in the DOX group compared with the control group, 7 proteins were upregulated in the DOX + CDC20 group compared with the DOX group, 104 proteins were upregulated in the DOX group compared with the control group, and 9 proteins were downregulated in the DOX + CDC20 group compared with the DOX group (Supplementary Fig. S8a). We identified CCDC69 and plotted its location on a volcano plot (Supplementary Fig. S8b). We found that DOX could enhanced the expression of CCDC69 in NRCMs and mouse hearts. Compared with that in the DOX group, CCDC69 expression was significantly decreased in the DOX + Ad/AAV9-CDC20 group, while it significantly increased in the DOX + Ad/AAV9-siCDC20 group. We speculated that CDC20 may ubiquitinate CCDC69 (Supplementary Fig. S8c–f). qPCR experiments demonstrated that CDC20 did not affect the mRNA expression level of CCDC69 (Supplementary Fig. S9).

**Fig. 5:**
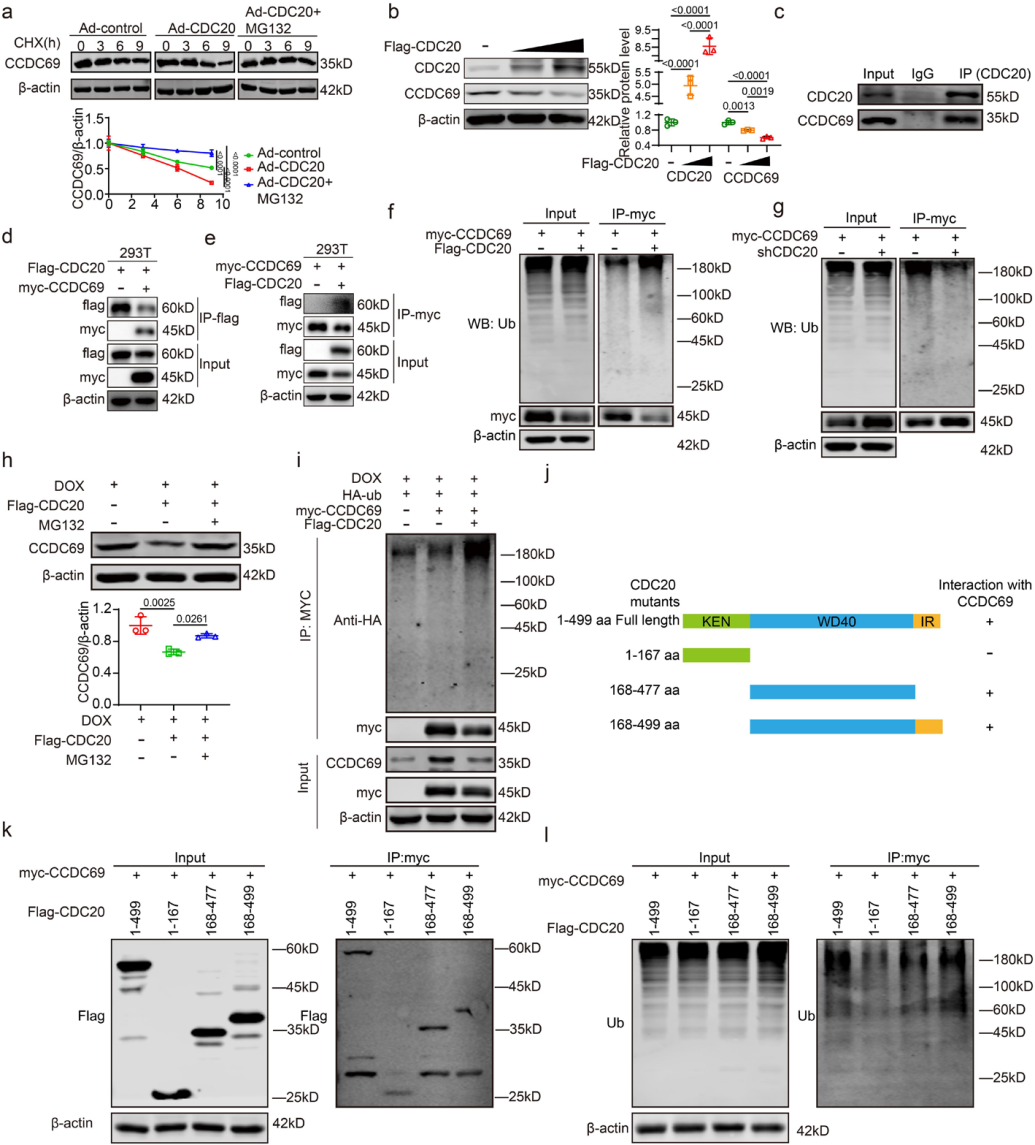
CDC20 is capable of ubiquitinating CCDC69 in cardiomyocytes. **a** NRCMs were infected with adenovirus Ad-GFP and Ad-CDC20, treated with CHX and/or MG132, and harvested at the specified time points (*n* = 3); **b** CDC20 expression through western blotting after transfecting different concentrations of CDC20 plasmid into 293 T cells (*n* = 3); **c** Using Western blot detected the expression of CDC20 and CCDC69 by co-IP; **d** Protein lysates were immunoprecipitated with an anti-Flag antibody and analyzed by western blotting using Flag, Myc, and β-actin (*n* = 3); **e** Protein lysates were immunoprecipitated with an anti-Myc antibody and analyzed by western blotting using Flag, Myc, and β-actin (*n* = 3); **f**, **g** Lysates harvested from AC16 cells after transfecting myc-CCDC69 and Flag-CDC20, pretreated with MG132 for 6 h, and immunoprecipitated with anti-myc. The ubiquitin-conjugated CCDC69 was detected by Western blotting using anti-ubiquitin (Ub). Input shows the expression of the corresponding proteins in whole-cell lysates (*n* = 3); **h** CCDC69 and β-actin expression through western blotting (*n* = 3). **i** Transfection with myc-CCDC69, Flag-CDC20, and HA-UB, followed by immunoprecipitation with anti-myc. The ubiquitin-conjugated CCDC69 was detected by western blotting (*n* = 3); **j** Schematic diagram depicting the domain structure of CDC20 and a summary of in vitro binding experiments (*n* = 3); **k** Western blot showing the expression of CDC20 mutant proteins tagged with Flag (*n* = 3); **l** Western blot showing the expression of ubiquitin after transfecting CDC20 mutant plasmid (*n* = 3). NRCM: neonatal rat cardiomyocyte. Data presented as mean ± SD. Statistical analysis performed with one-way ANOVA or two-way ANOVA

Experiments in which NRCMs were stimulated with cycloheximide (CHX) and MG132 revealed that CDC20 overexpression reduced the half-life of CCDC69, a process that could be reversed by the addition of MG132 (Fig. 5a). In 293 T cells, increasing the concentration of the transfection plasmids resulted in enhanced degradation of CCDC69 upon CDC20 overexpression (Fig. 5b). Co-IP was performed in 293 T. The results showed that CCDC69 was efficiently precipitated by the anti-CDC20 antibody but not by an IgG control (Fig. 5c). Co-transfection of Flag-CDC20 and myc-CCDC69 in 293 T cells followed by immunoprecipitation with anti-Flag and anti-Myc antibodies revealed a potential interaction between CDC20 and CCDC69 (Fig. 5d, e). To assess the ubiquitination of CCDC69 by CDC20, AC16 cells were transfected with Flag-CDC20 and myc-CCDC69, followed by immunoprecipitation using a Myc antibody. Immunoblotting revealed that CDC20 overexpression markedly enhanced the ubiquitination of CCDC69. In contrast, transfection of siCDC20 plasmids and myc-CCDC69, followed by immunoprecipitation using an anti-Myc antibody, revealed that interference with CDC20 substantially suppressed the ubiquitination of CCDC69 (Fig. 5f, g).

Next, AC16 cells were stimulated with DOX, MG132, and Flag-CDC20. These results indicate that the addition of MG132 inhibited the degradation of CCDC69 by CDC20 (Fig. 5h). After myc-CCDC69 transfection, the overexpression of CDC20 induced by DOX treatment increased the ubiquitination level of CCDC69 (Fig. 5i). To identify the specific domain of CDC20 involved mediating the ubiquitination of CCDC69, we transfected full-length FLAG-tagged CDC20 and various CDC20 mutants into 293 T cells (Fig. 5j, k). Immunoblotting using anti-Flag and anti-Ub showed that two segments of CDC20, amino acids 168–477 and 168–499, clearly mediated CCDC69 ubiquitination, while The amino acids 1–167 fragment weakly mediated the ubiquitination of CCDC69 (Fig. 5l). These findings suggested that CDC20 directly ubiquitinates CCDC69 in myocardial cells.

### Overexpression of CCDC69 intervenes in the protective effect of CDC20 against DOX-induced myocardial injury

**Fig. 6:**
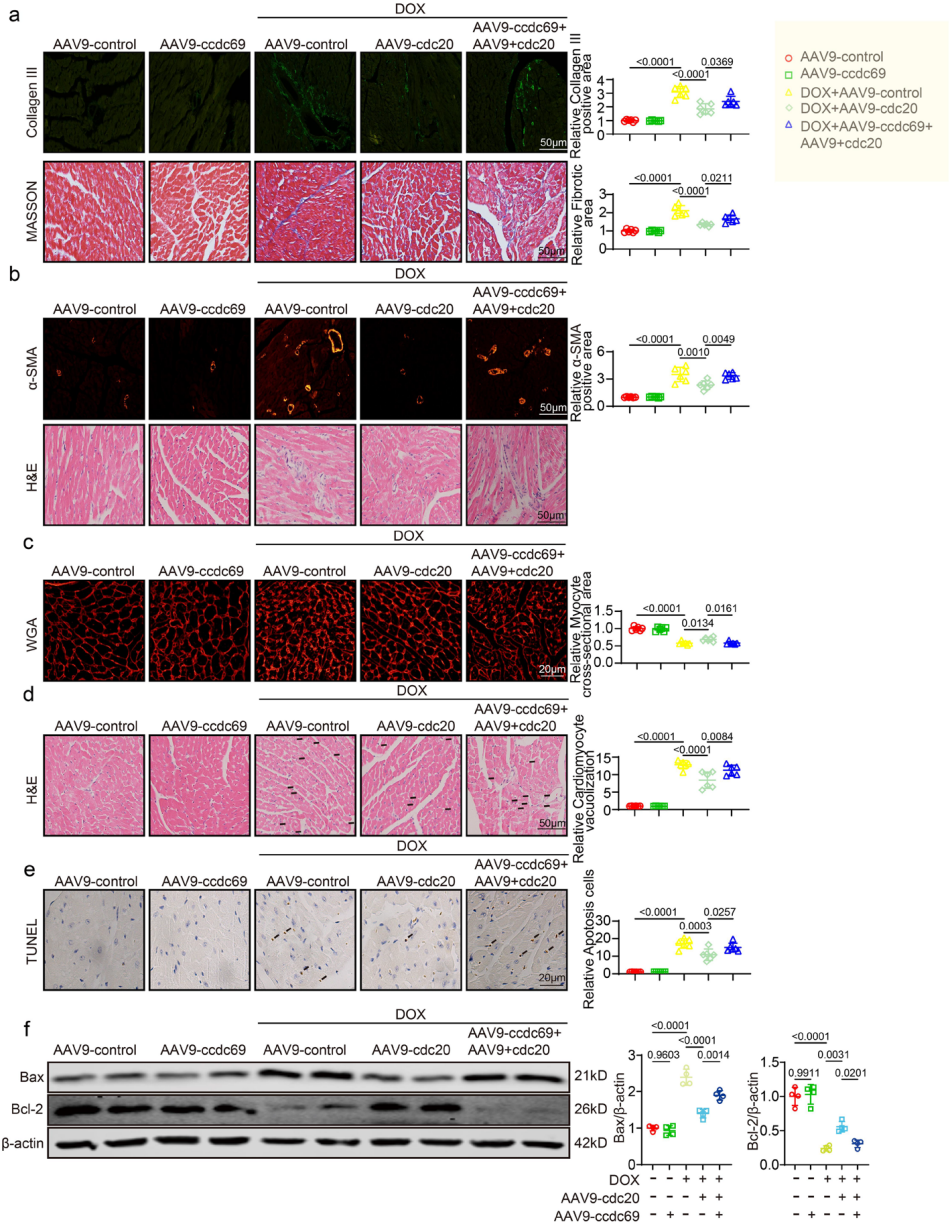
Overexpression of CCDC69 intervenes in the protective effect of CDC20 against doxorubicin-induced myocardial injury. **a** Representative images and statistical data of Masson and collagen III staining in each group (*n* = 6, bar = 50 μm); **b** Representative images and statistics data of α-SMA and H&E staining in each group (*n* = 6, bar = 50 μm); **c** Representative images and statistical data of WGA staining in each group (*n* = 6, bar = 20 μm); **d** Representative images and statistical data of vacuolization in each group (*n* = 6, bar = 50 μm); **e** Representative images and statistical data of TUNEL staining in each group (*n* = 6, bar = 20 μm); **f** Bax, Bcl-2, and β-actin expression through western blot (*n* = 4). α-SMA: alpha-smooth muscle actin, H&E: hematoxylin and eosin, TUNEL: terminal deoxynucleotidyl transferase dUTP nick end labelling, WGA: wheat germ agglutinin. Data presented as mean ± SD. Statistical analysis performed with two-way ANOVA

Next, we conducted in vivo experiments to investigate the potential role of CCDC69 in mediating the anti-DIC effects of CDC20. Using Masson, H&E, and IF (Collagen III and α-SMA) staining, we confirmed that CCDC69 overexpression inhibited the extent of DOX-induced myocardial fibrosis and inflammation mediated by CDC20 (Fig. 6a, b). WGA staining revealed that CCDC69 overexpression suppressed the degree of DOX-induced myocardial atrophy mediated by CDC20 (Fig. 6c). H&E staining indicated that CCDC69 overexpression inhibited the extent of DOX-induced myocardial vacuolization mediated by CDC20 (Fig. 6d). TUNEL and western blotting experiments demonstrated that CCDC69 overexpression suppressed DOX-induced apoptosis mediated by CDC20 (Fig. 6e, f). When we overexpressed both CDC20 and CCDC69 plasmids in AC16 cells, CCDC69 overexpression inhibited DOX-induced apoptosis by mediated by CDC20 (Supplementary Fig. S8g). Together, these findings demonstrated that overexpression of CCDC69 intervened in the DOX-induced myocardial injury-protective effect of CDC20.

### Cardiomyocyte-specific overexpression of CDC20 significantly inhibits DOX-induced myocardial injury, not affecting the antitumor effect of DOX

**Fig. 7:**
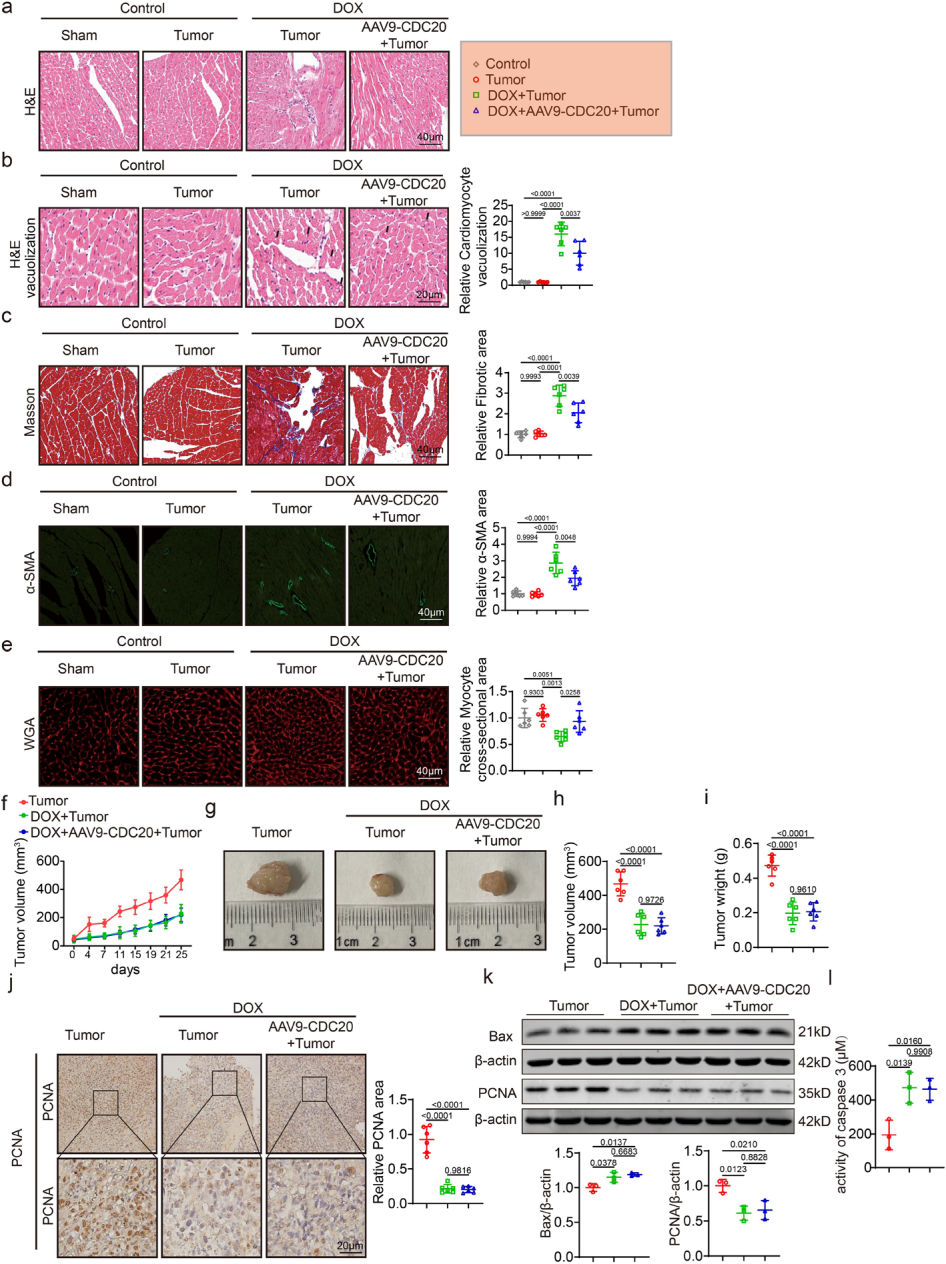
Cardiomyocyte-specific overexpression of CDC20 significantly inhibits DOX-induced myocardial injury, while not affecting the antitumor effect of DOX. **a** Representative images of HE staining in each group (*n* = 6, bar = 100 μm); **b** Representative images and statistical data of vacuolization in each group (*n* = 6, bar = 50 μm); **c** Representative images and statistical data of Masson staining in each group (*n* = 6, bar = 100 μm); **d** Representative images and statistical data of α-SMA staining in each group (*n* = 6, bar = 50 μm); **e** Representative images and statistical data of WGA staining in each group (*n* = 6, bar = 50 μm); **f** Tumor growth curves of mice in each group (*n* = 6); **g** Representative images of tumor in each group; **h** Statistical data of tumor volume (*n* = 6); **i** Statistical data of tumor weight (*n* = 6); **j** Representative images and statistical data of PCNA staining in each group (*n* = 6, bar = 50 μm); **k** Bax, PCNA, and β-actin expression through western blot (*n* = 3); **l** Caspase-3 activity in each group (*n* = 3). α-SMA: alpha-smooth muscle actin, H&E: hematoxylin and eosin, HW/TL: heart weight/tibia length, PCNA: proliferating cell nuclear antigen, TUNEL: terminal deoxynucleotidyl transferase dUTP nick end labeling, WGA: wheat germ agglutinin. Data presented as mean ± SD. Statistical analysis performed with two-way ANOVA

We found that cardiomyocyte-specific CDC20 overexpression inhibited DOX-induced myocardial injury; however, it remained unclear whether it would affect the antitumor effects of DOX. Tumor-bearing mice were created by injecting Panc02 cells 2 weeks after AAV9-cTNT-CDC20 administration via the tail vein. One week later, DOX (5 mg/kg per week) was administered into the tail vein. The total DOX dose was 20 mg/kg/week, 4 weeks. We first assessed cardiac indicators in each group. Inflammatory aggregation and vacuolation were evaluated by H&E staining of mouse hearts. The extent of cardiac inflammatory cell infiltration and injury were not affected in tumor-bearing mice. However, DOX administration led to an increase in cardiac inflammatory aggregates and vacuolation compared with those in the tumor group, whereas CDC20 overexpression suppressed these effects of DOX (Fig. 7a, b). Masson and IF (α-SMA) staining analyses revealed that DOX administration led to cardiac fibrosis, which was significantly decreased after CDC20 overexpression (Fig. 7c, d). WGA staining indicated that DOX administration caused cardiomyocyte atrophy, whereas cardiomyocytes size increased after CDC20 overexpression compared with the DOX + tumor group (Fig. 7e).

DOX was administered when the tumors reached a size of approximately 100 mm^3^. Tumor volume was measured using sliding calipers at 2-day intervals. As shown in Fig. 7f and Fig. 7h, tumor volumes were significantly decreased in both the DOX + tumor and DOX + AAV9-CDC20 + tumor groups compared with the tumor group, with no difference between the DOX + tumor and DOX + AAV9-CDC20 + tumor groups. After 3 weeks, the mice were euthanized, and findings revealed that tumor weights were significantly decreased in both the DOX + tumor and DOX + AAV9-CDC20 + tumor groups compared with the tumor group, with no difference between the former two groups (Fig. 7g, i). To validate these findings, we employed immunohistochemistry (proliferating cell nuclear antigen [PCNA]) and western blotting to detect apoptosis- and proliferation-related proteins. The results demonstrated notable upregulation of BAX in both the DOX + tumor group and the DOX + AAV9-CDC20 + tumor groups compared with the tumor group. Conversely, PCNA was notably downregulated in these two groups compared with the tumor group (Fig. 7j, k). DOX increased caspase 3 activity in in both the DOX + tumor group and the DOX + AAV9-CDC20 + tumor groups compared with the tumor group (Fig. 7l). Using the same method, we have also demonstrated in melanoma that cardiomyocyte-specific overexpression of CDC20 significantly inhibits DOX-induced myocardial injury, not affecting the antitumor effect of DOX (Supplementary Fig. S10).

## Discussion

Since its discovery in the 1950s and medical approval in the USA in 1974, DOX has been utilized for the treatment of various cancers, including breast cancer, lymphoma, acute lymphoblastic leukemia, bladder cancer, and melanoma. DOX is intravenously injected in cancer patients. However, mounting evidence indicates that DOX treatment can lead to heart failure and severe cardiomyocyte apoptosis, both of which significantly contribute to the development of cardiac dysfunction and associated complications in clinical patients and preclinical mouse models [16, 17]. Despite their critical importance, the mechanisms responsible for the excessive cardiomyocyte apoptosis during chemotherapy remain largely unknown. Hence, the significance of anti-apoptotic effects in the treatment of DOX-related cardiotoxicity cannot be overstated [18]. This study aimed to identify anti-apoptotic genes using transcriptomics. Significant decreases in CDC20 expression have been observed after DOX treatment. Owing to ongoing advancements in clinical molecular biology, CDC20 has been identified as a crucial contributor to the initiation and progression of malignant tumors, primarily because of its elevated expression levels. Elevated CDC20 expression in tumors promotes cell growth, suppresses apoptosis, and facilitates cell migration and invasion [19–21]. Therefore, we hypothesized that CDC20 exerts an anti-apoptotic effect on cardiomyocytes. Our previous study demonstrated that CDC20 overexpression exacerbated transverse aortic constriction-induced cardiac hypertrophy and cardiomyocyte enlargement in mice [12]. As DOX reduces cardiomyocytes size, we investigated whether CDC20 could counteract this effect. In the current study, we discovered that targeted myocardial overexpression of CDC20 using AAV9 suppressed DOX-induced apoptosis in cardiomyocytes. Suppression of CDC20 expression exacerbated DOX-induced cardiomyocyte apoptosis. Experiments using the CDC20 inhibitor apcin [11, 22], AAV9-siCDC20, and cardiomyocyte-specific CDC20 knockout models further supported our conclusion that inhibiting CDC20 exacerbates cardiomyocyte apoptosis and increases mouse mortality.

Using proteomic analysis, we discovered that CDC20 targets CCDC69 to inhibit cardiomyocyte apoptosis. CCDC proteins contain a versatile coiled-coil motif and play roles in regulating the cell cycle and apoptosis [23, 24]. CCDC69, which is located at position 5q33.1, is a member of this family [13]. Additionally, CCDC69 plays a role in controlling central spindle assembly and recruiting intermediate-region components that are essential for cytoplasmic division in animal cells. CCDC69 is typically expressed at low levels in tumor tissues [25]. CCDC69 has been shown to enhance platinum-induced apoptosis in ovarian cancer cells and to increase their sensitivity to combination therapy with platinum drugs [26]. CCDC69 overexpression activates the p14ARF/MDM2/p53 pathway in ovarian cancer, leading to improved cisplatin resistance [15]. Through in vitro experiments, we confirmed that CDC20 ubiquitinates CCDC69 and that CCDC69 inhibits the anti-DIC effect of CDC20.

DOX belongs to the anthracycline class of antibiotics and is a widely used anticancer drug. However, its long-term use may lead to cardiotoxicity, particularly by inducing myocardial apoptosis [27]. DOX induces apoptosis in cardiomyocytes via various pathways, including binding to DNA and interfering with its replication and repair, resulting in DNA damage and eventually apoptosis [28]. DOX can also cause mitochondrial dysfunction, leading to the loss of mitochondrial membrane potential and the release of apoptosis-related proteins such as cytochrome C, thereby activating the apoptotic pathway [29]. Myocardial apoptosis causes irreversible damage to cardiac function and may result in cardiac dysfunction. Bax and Bcl-2 are two important Bcl-2 family members that play critical roles in regulating cell apoptosis [30]. Bax is a pro-apoptotic protein that increases mitochondrial membrane permeability, leading to the release of apoptosis-related proteins from the mitochondria into the cytoplasm, thereby triggering apoptosis [31]. In contrast, Bcl-2 is an anti-apoptotic protein that inhibits Bax activity and maintains mitochondrial membrane integrity, thereby preventing apoptosis. The Bax/Bcl-2 balance plays an important regulatory role in myocardial apoptosis [32]. An increase in Bax expression/ activity or a decrease in Bcl-2 expression/activity may elevate the risk of myocardial cell apoptosis. We demonstrated that CDC20 inhibited DOX-induced Bax expression and suppressed Bcl-2 expression. Furthermore, in both cell and animal experiments, overexpression of CCDC69 inhibited the anti-DIC effect of CDC20. Therefore, we conclude that CDC20 inhibits CCDC69 expression, thereby mitigating DOX-induced damage to cardiomyocytes.

To investigate the potential myocardial injury caused by DOX inhibition, while preserving its antitumor effect [33], we comprehensively investigated the role of CDC20 in mice bearing tumors. Our findings suggested that CDC20 overexpression, particularly in myocardial cells, could effectively inhibit DOX-induced cardiomyocyte apoptosis, while also inhibiting pancreatic carcinoma and melanoma growth. Previous studies have shown that CDC20 is significantly upregulated in tumor tissues. However, the use of a CDC20 agonist for heart protection via intravenous injection may compromise the antitumor effects of DOX, whereas CDC20 inhibitors exacerbate myocardial damage. We successfully demonstrated that AAV9 provides an effective alternative.

AAV9 offers several advantages, including notable safety, stable expression, and strong targeting ability. Currently, recombinant AAV is primarily used in clinical applications for gene therapies for rare, ophthalmic, metabolic, cardiovascular, and nervous system diseases [34]. In a porcine model of myocardial infarction, researchers used AAV9 gene therapy to locally knockdown the Hippo pathway gene salvador (Sav) in border-zone cardiomyocytes. Additionally, AAV9-Sav-shRNA treatment resulted in a reduction in heart scar size in pigs with myocardial infarction. AAV9-Sav-shRNA gene therapy was well tolerated and did not result in death. Furthermore, histopathological examination of the liver and lungs revealed no tumor formation [35]. The use of AAV to deliver MOG1 has been suggested as an alternative therapy for treating Brugada syndrome resulting from SCN5A gene mutations. The study by Smith et al. introduces a therapeutic strategy focusing on chaperone proteins responsible for regulating protein trafficking, offering an effective treatment for sodium channel diseases [36].

At present, there have been clinical trials of AAV-SERCA2a in the treatment of myocardial failure, but the results show that it cannot reduce recurrence and prolong the time to end-stage events. However, in clinical trials of other diseases, such as Hunyenton’s disease, severe hemophilia, and vascular disease macular degeneration, AAV treatment has achieved good results. It can be seen that there is still hope for AAV to treat heart disease, although perhaps the method is wrong. In this study, we used the tail vein method of AAV9-cTNT-CDC20 to prove that overexpression of CDC20 can inhibit DOX-induced myocardial injury in mice, thereby reducing the development of heart failure in mice, and without affecting the antitumor effect of DOX.

In recent years, great changes have taken place in the field of tumor treatment, which has significantly improved the long-term prognosis of tumor patients. However, with the extension of treatment time and survival time, and the wide application of targeted drugs, there are more and more studies on complications such as heart failure, hypertension, thromboembolism, and arrhythmia caused by drugs. Cardiovascular disease has become the second leading cause of death in patients with recurrent metastatic tumors. For cancer patients, clinicians not only pay attention to the effectiveness of cancer treatment, but also pay attention to the prevention and treatment of cardiovascular complications and complications. If we successfully develop AAV9-cTNT-CDC20 and industrialize the project products according to the current medical and scientific technology, it will not only have a broad sales market, but also have great social significance and economic value for improving the quality of life of patients with heart failure and promoting their health and rehabilitation.

## Conclusions

Our study provides valuable insights into the role of CDC20 in DIC and contributes new information on potential molecular mechanisms and pathways that underpin DIC, which may lead to risk mitigation and the development and refinement of effective cancer therapies. We believe that our research findings can serve as guidance for appropriate and timely treatment strategies that can be tailored to specific patient needs and improve the clinical management of DIC.

## Supplementary Information

Additional file 1.

## Data Availability

All data will be shared upon reasonable request to the corresponding author.

## Abbreviations

DOX: Doxorubicin
DIC: DOX-induced cardiotoxicity
CDC20: Cell division cycle 20
CCDC69: Coiled-coil domain containing 69
OS: Overall survival
DEGs: Differentially expressed genes
DEPs: Differentially expressed proteins
EF: Ejection fraction
FS: Fractional shortening
H&E: Hematoxylin–eosin
SDS-PAGE: Sodium dodecyl sulfate–polyacrylamide gel electrophoresis
PVDF: Polyvinylidene fluoride
FBS: Fetal bovine serum
co-IP: Co-immunoprecipitation
SD: Standard deviation
HW/TL: Heart weight/tibia length
NRCM: Neonatal rat cardiomyocytes
GO: Gene Ontology
KEGG: Kyoto Encyclopedia of Genes and Genomes
Ad-CDC20: Adenovirus overexpressing CDC20
AAV: Adeno-associated virus
WGA: Wheat germ agglutini
TUNEL: Transferase dUTP nick end labeling
CHX: Cycloheximide
PCNA: Proliferating cell nuclear antige
Sav: Salvador
