# Correction: CDC20 protects the heart from doxorubicin-induced cardiotoxicity by modulating CCDC69 degradation

**DOI:** 10.1186/s11658-025-00767-x

**Published:** 2025-07-19

**Authors:** Zhenyu Feng, Ningning Zhang, Liang Wang, Xumin Guan, Yunpeng Xie, Yun-long Xia

**Affiliations:** 1 Institute of Cardiovascular Diseases, The First Affiliated Hospital of Dalian Medical University, Lianhe Road 193, Dalian, 116000 Liaoning People’s Republic of China; 2 Department of Hematology, The First Affiliated Hospital of Dalian Medical University, Dalian, People’s Republic of China; 3 Department of Pharmacy, Liaoyang City Central Hospital, Liaoyang, People’s Republic of China

**Correction: Cellular & Molecular Biology Letters (2025) 30:29** 10.1186/s11658-025-00708-8

Following publication of the original article [1], the authors regret that the original version of this paper unfortunately contained an incorrect image in Figure S10B. The Figures used in the DOX  + Tumor groups were repeated. We have double checked the original data and found that the inadvertent errors occurred during figure compilation, and this correction does not affect the scientific conclusions of the article.

The correct Figure S10B is given in this correction.
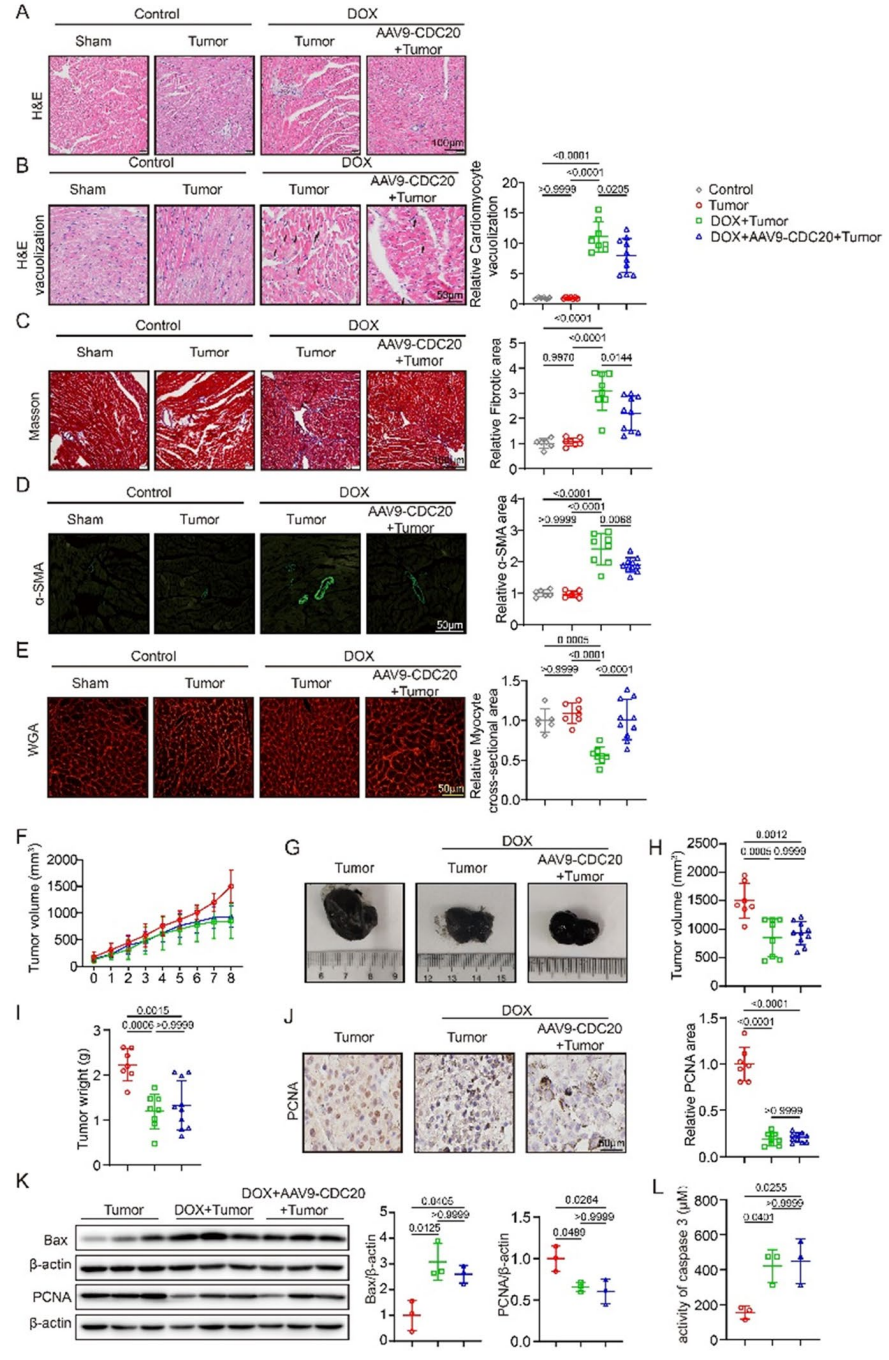


Fig. S10 Cardiomyocyte-specific overexpression of CDC20 significantly inhibits DOX-induced myocardial injury, while not affecting the antitumor effect of DOX. (a) Representative images of HE staining in each group (n = 6–10, bar = 100 μm); (b) Representative images and statistical data of vacuolization in each group (n = 6–10, bar = 50 μm); (c) Representative images and statistical data of Masson staining in each group (n = 6–10, bar = 100 μm); (d) Representative images and statistical data of α-SMA staining in each group (n = 6–10, bar = 50 μm); (e) Representative images and statistical data of WGA staining in each group (n = 6–10, bar = 50 μm); (f) Tumor growth curves of mice in each group (n = 7–10); (g) Representative images of tumor in each group; (h) Statistical data of tumor volume (n = 7–10); (i) Statistical data of tumor weight (n = 7–10); (j) Representative images and statistical data of PCNA staining in each group (n = 7–10, bar = 50 μm); (k) Bax, PCNA and β-actin expression through western blot (n = 3); (l) Caspase-3 activity in each group (n = 3). α-SMA: alpha-smooth muscle actin, H&E: Hematoxylin and Eosin, HW/TL: heart weight/tibia length, PCNA: Proliferating Cell Nuclear Antigen, TUNEL: terminal deoxynucleotidyl transferase dUTP nick end labelling, WGA: wheat germ agglutinin. Data are represented as the mean ± SD. Statistical analysis was performed with two-way ANOVA
