# Supplementary material for: CDC20 protects the heart from doxorubicin-induced cardiotoxicity by modulating CCDC69 degradation
Source: Cell Mol Biol Lett. 2025 Mar 5;30:29. doi: 10.1186/s11658-025-00708-8 (PMC11884132; doi:10.1186/s11658-025-00708-8)
Supplement: Supplementary file 1 — Additional file 1. [file 11658_2025_708_MOESM1_ESM.docx]

**Supplementary Figures and Figure legends**


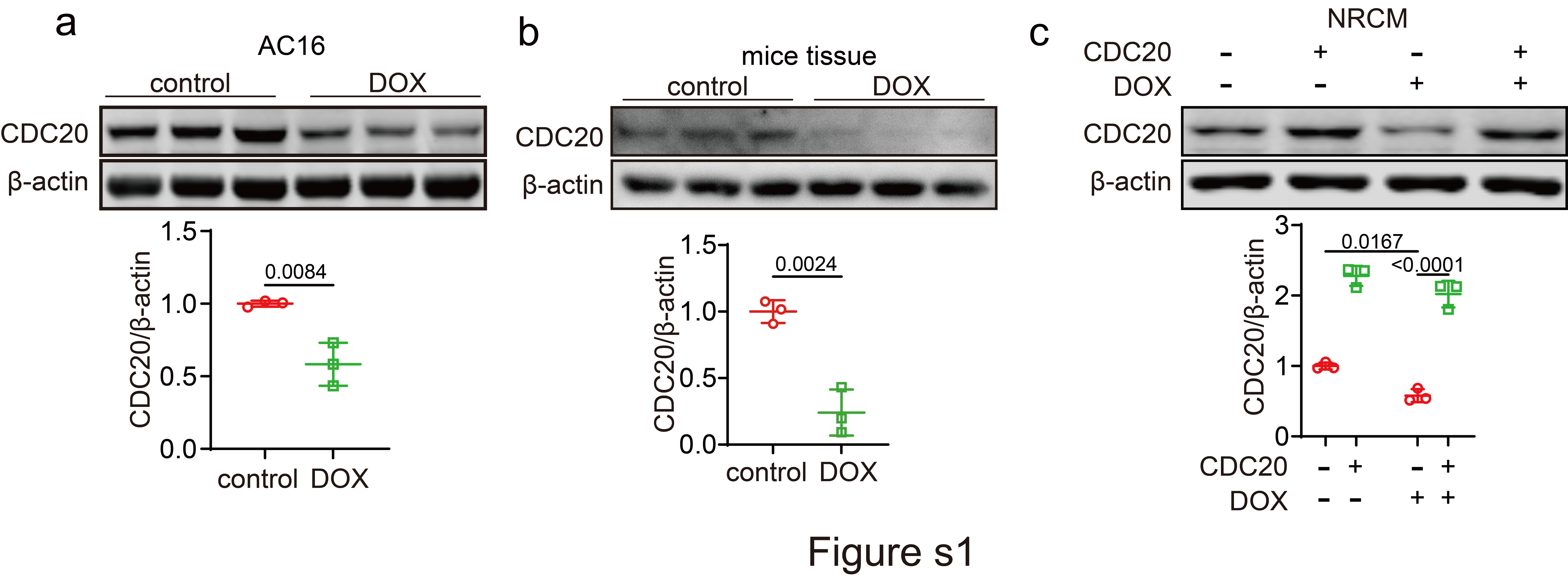


Fig. S1 DOX inhibits CDC20 expression in cardiac myocytes. (a) Expression of CDC20 in AC16 cells treated with DOX (n=3); (b) CDC20 expression in mouse hearts treated with DOX (n=3); (c) CDC20 expression in NRCMs treated with DOX (n=3). DOX: Doxorubicin, NRCM: neonatal rat cardiomyocyte. Data are represented as the mean ± SD. Statistical analysis was performed with Student’s t-test or two-way ANOVA.


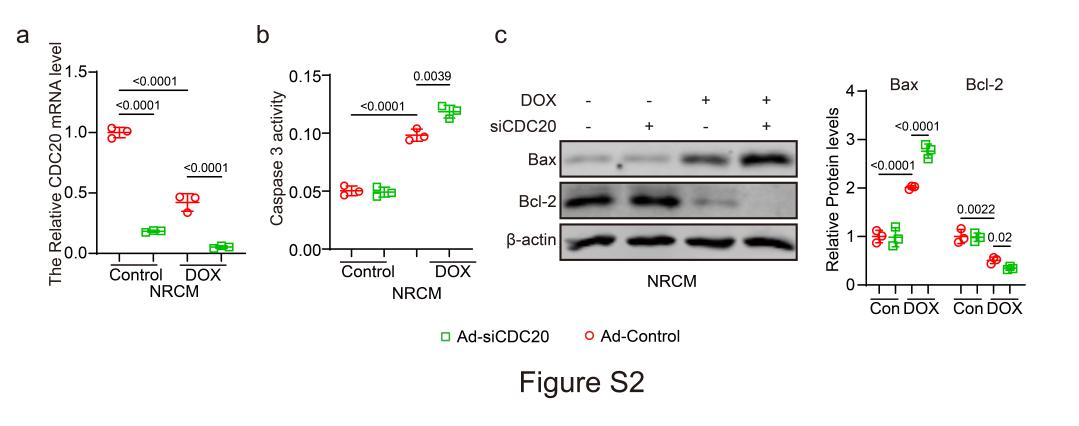


Fig. S2 CDC20 interference increases Dox-induced apoptosis in myocardial cells (a) mRNA expression of CDC20 was detected in NRCMs treated with doxorubicinv; (b) Caspase 3 activity was measured in NRCMs treated with DOX and Ad-siCDC20 (n=3); (c)The expression of Bax, Bcl-2, and β-actin was examined in NRCMs treated with DOX and Ad-siCDC20 (n=3). DOX: Doxorubicin, NRCM: neonatal rat cardiomyocyte. Data are represented as the mean ± SD. Statistical analysis was performed with two-way ANOVA.


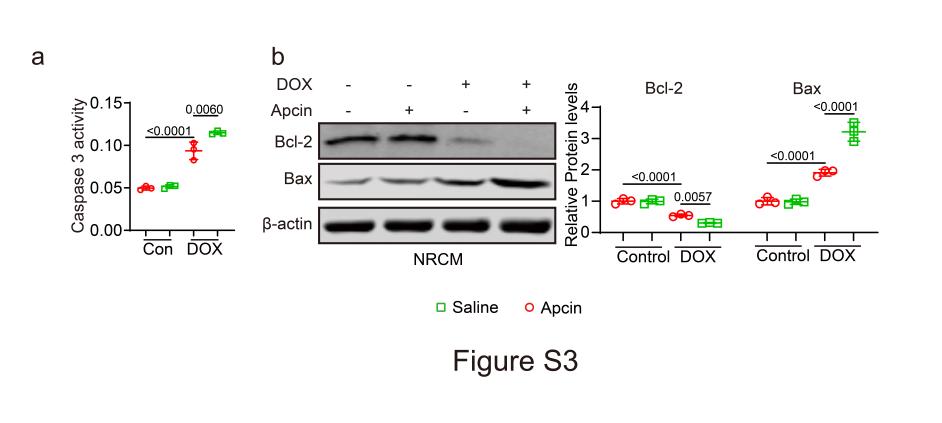


Fig. S3 Apcin, a CDC20 inhibitor, exacerbates DOX-induced myocardial cell apoptosis. (a) Caspase 3 activity in NRCMs treated with DOX and Ad-siCDC20 (n=3); (b) Bax, Bcl-2, and β-actin expression in NRCMs treated with DOX and Ad-siCDC20 (n=3). DOX: Doxorubicin, NRCM: neonatal rat cardiomyocyte. Data are represented as the mean ± SD. Statistical analysis was performed with two-way ANOVA.


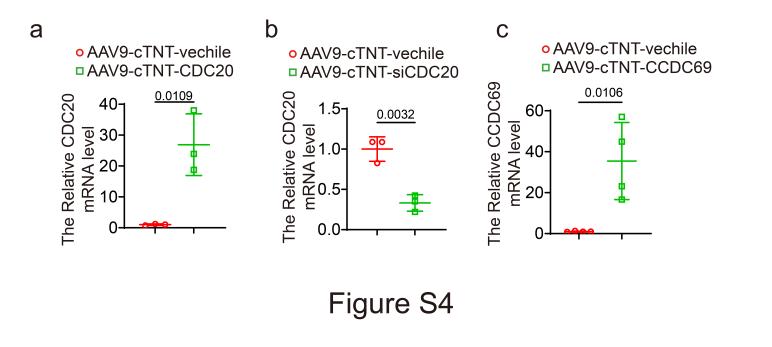


Fig. S4 Detection of the infection efficiency of three AAV9 viruses. (a) mRNA expression of CDC20 in mice heart treated with AAV9-cTNT-CDC20 (n=3); (b) mRNA expression of CDC20 in mice heart treated with AAV9-cTNT-siCDC20 (n=3); (c) mRNA expression of CCDC69 in mice heart treated with AAV9-cTNT-CCDC69 (n=3). AAV9: Adeno-Associated Virus 9. Data are represented as the mean ± SD. Statistical analysis was performed with Student’s t-test.


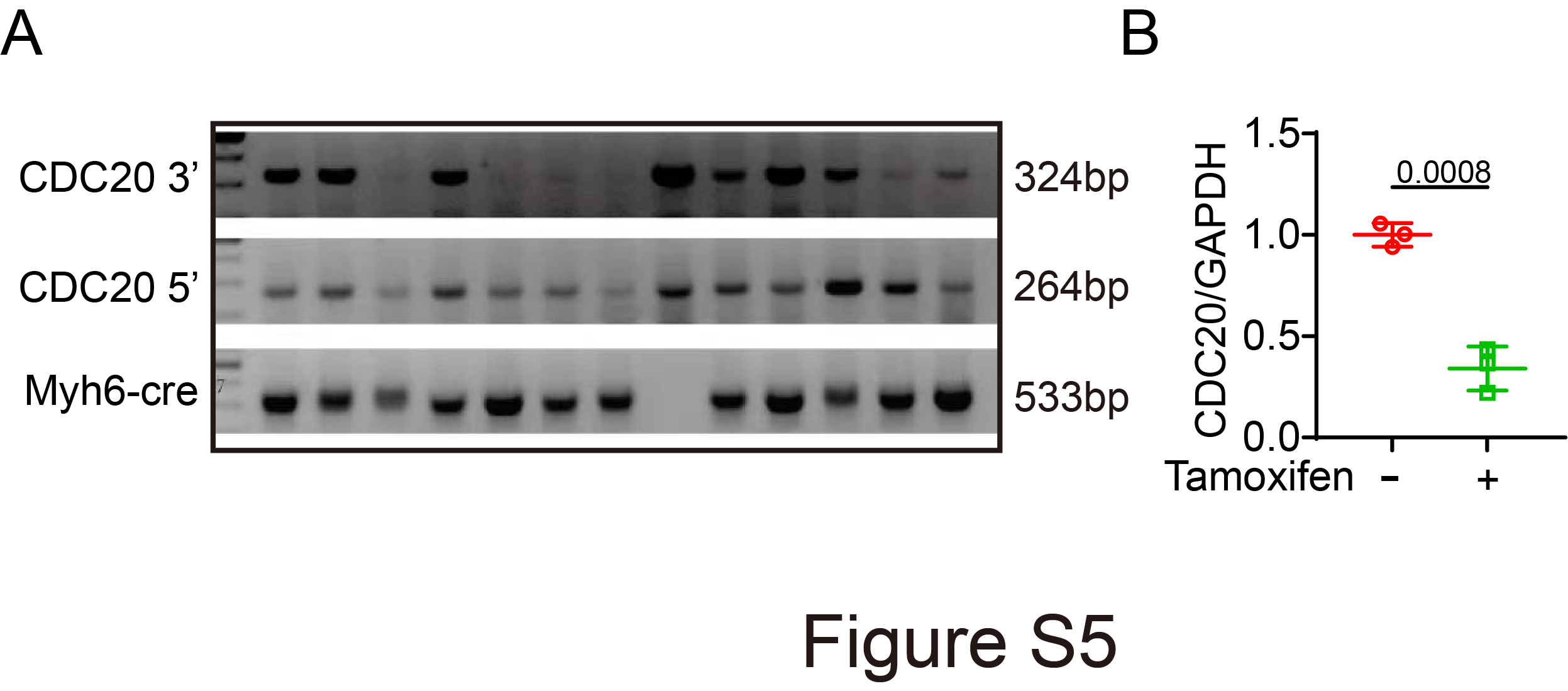


Fig. S5 Infection efficiency of myocardium-specific CDC20 knockout mice. (a) Agarose gel electrophoresis was performed to show the genotyping results of mouse CDC20. (b) mRNA expression of CDC20 in mice heart treated with tamoxifen (n=3). Data are represented as the mean ± SD. Statistical analysis was performed with Student’s t-test


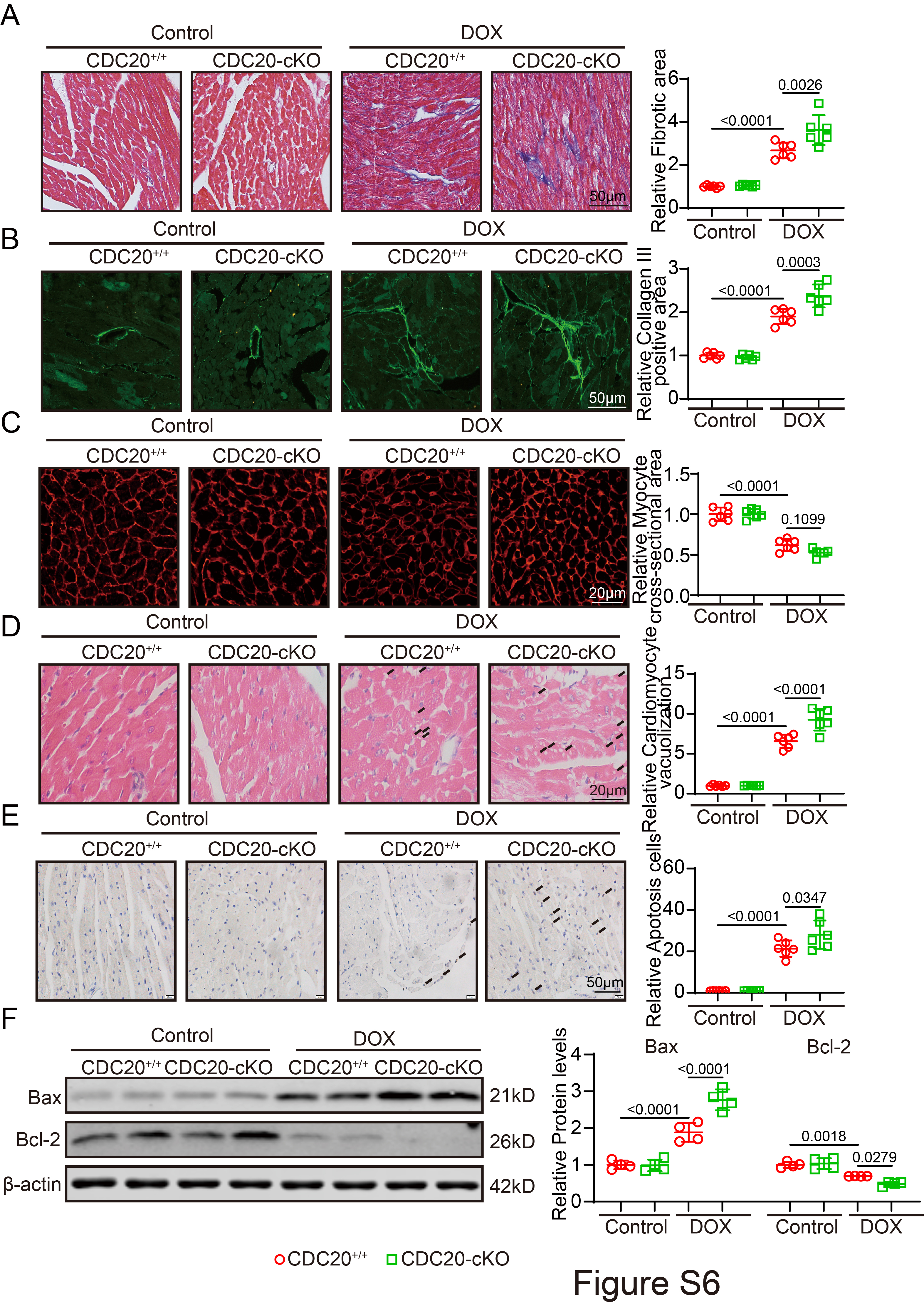


Fig. S6 AAV9-cTNT-siCDC20 exacerbates DOX-induced cardiac dysfunction, fibrosis, and apoptosis. (a) Representative images and statistical data of Masson staining in each group are presented (n=6, bar=50μm). (b) Representative images and statistical data of Collagen III staining in each group are presented (n=6, bar=50μm). (c) Representative images and statistical data of WGA staining in each group are presented (n=6, bar=20μm). (d) Representative images and statistical data of vacuolization in each group are presented (n=6, bar=20μm). (e) Representative images and statistical data of TUNEL staining in each group are presented (n=6, bar=50μm). (f) Bax, Bcl-2, and β-actin expression through western blotting (n=4). Data are represented as the mean ± SD. Statistical analysis was performed with two-way ANOVA.


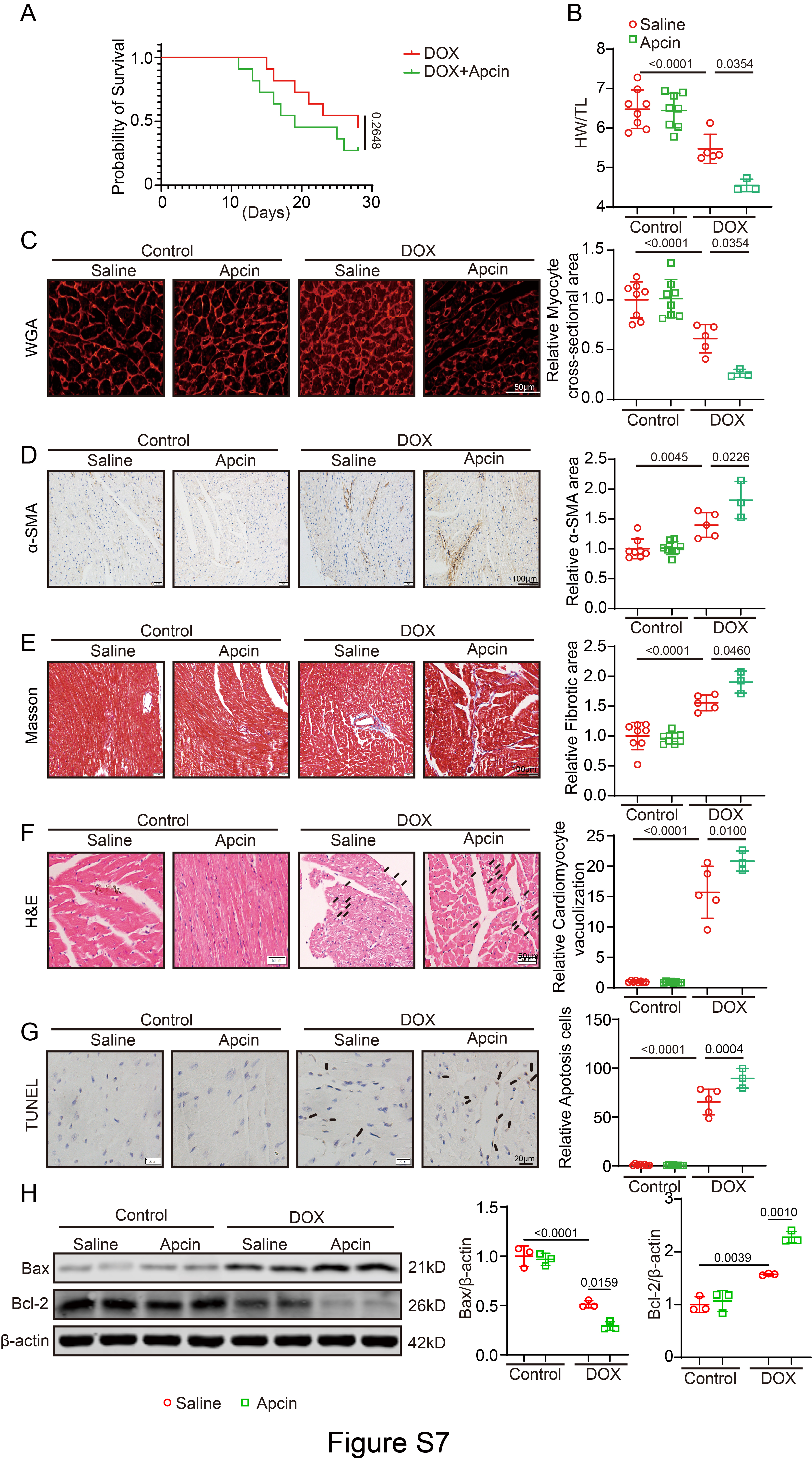


Fig. S7 Apcin exacerbates DOX-induced cardiac dysfunction, fibrosis, and apoptosis. (a) Survival curves of each mouse group. (b) Statistical data of HW/TL in each mouse group (n=3-8). (c) Representative images and statistical data of WGA staining in each group (n=3-8, bar=50μm). (d) Representative images and statistical data of α-SMA staining in each group (n=3-8, bar=100μm). (e) Representative images of Masson staining in each group (n=3-8, bar=100μm). (f) Representative images and statistical data of vacuolization in each group (n=3-8, bar=100μm). (g) Representative images and statistical data of TUNEL staining in the group (n=3-8, bar=20μm). (h) Expression of Bax, Bcl-2, and β-actin detected by western blot (n=3). α-SMA: alpha-smooth muscle actin, DOX: Doxorubicin, HW/TL: heart weight/tibia length, TUNEL: terminal deoxynucleotidyl transferase dUTP nick end labelling, WGA: wheat germ agglutinin. Data are represented as the mean ± SD. Statistical analysis was performed with two-way ANOVA.


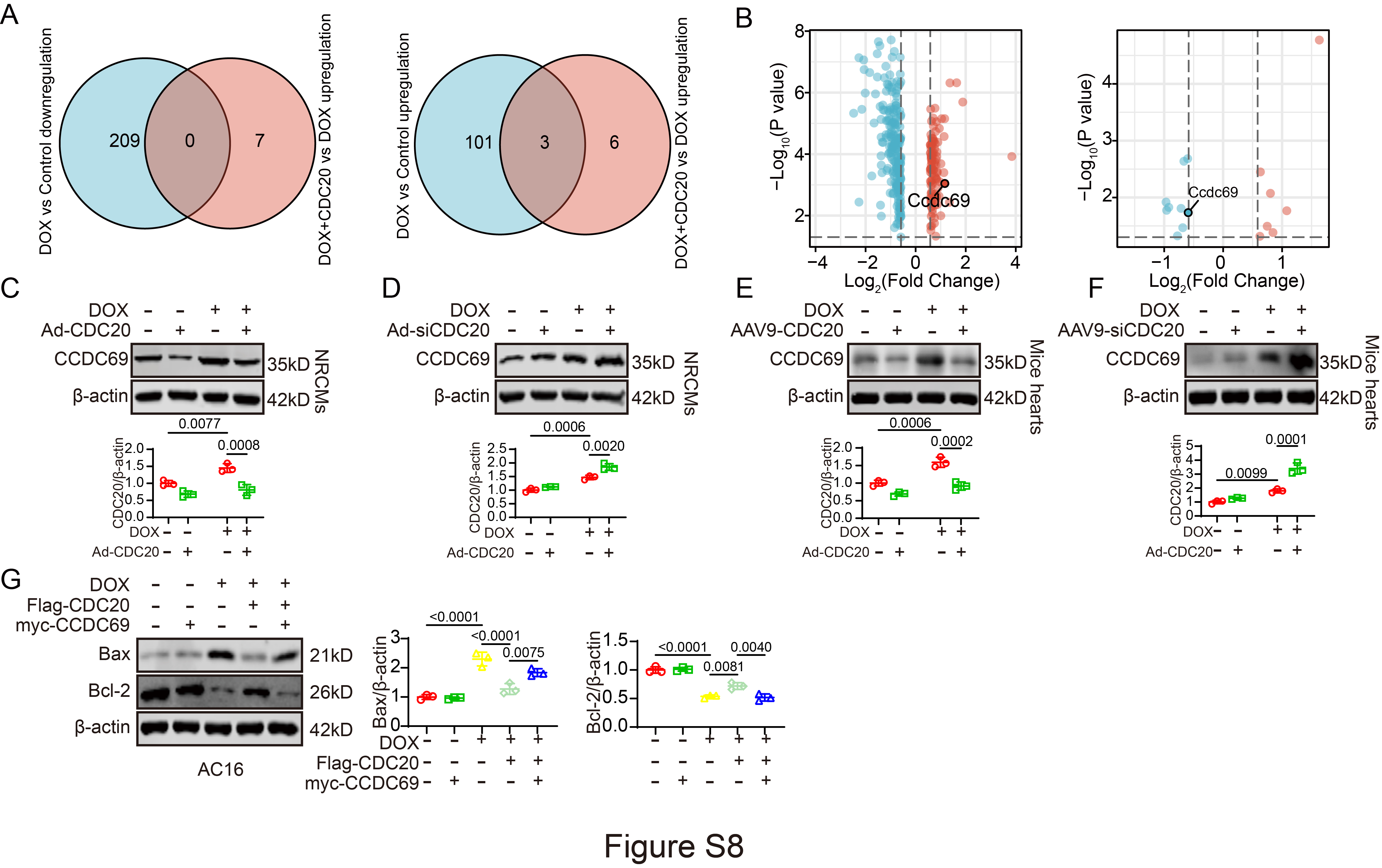


Fig. S8 CDC20 interferes with DOX-induced cardiomyocyte apoptosis by inhibiting CCDC69. (a) Venn diagram. (b) Volcano plot showing protein expression and the location of CCDC69 expression. (c, d) Detection of CCDC69 expression in NRCMs treated with DOX and adenovirus-CDC20/adenovirus-siCDC20 (n=3). (e, f) Detection of CCDC69 expression in the hearts of mice treated with DOX and AAV9-CDC20/AAV9-siCDC20 (n=3). (g) Detection of CCDC69 expression in AC16 cells treated with DOX, Flag-CDC20, and myc-ccdc69 (n=3). DOX: Doxorubicin, NRCM: neonatal rat cardiomyocyte. Data are represented as the mean ± SD. Statistical analysis was performed with two-way ANOVA.


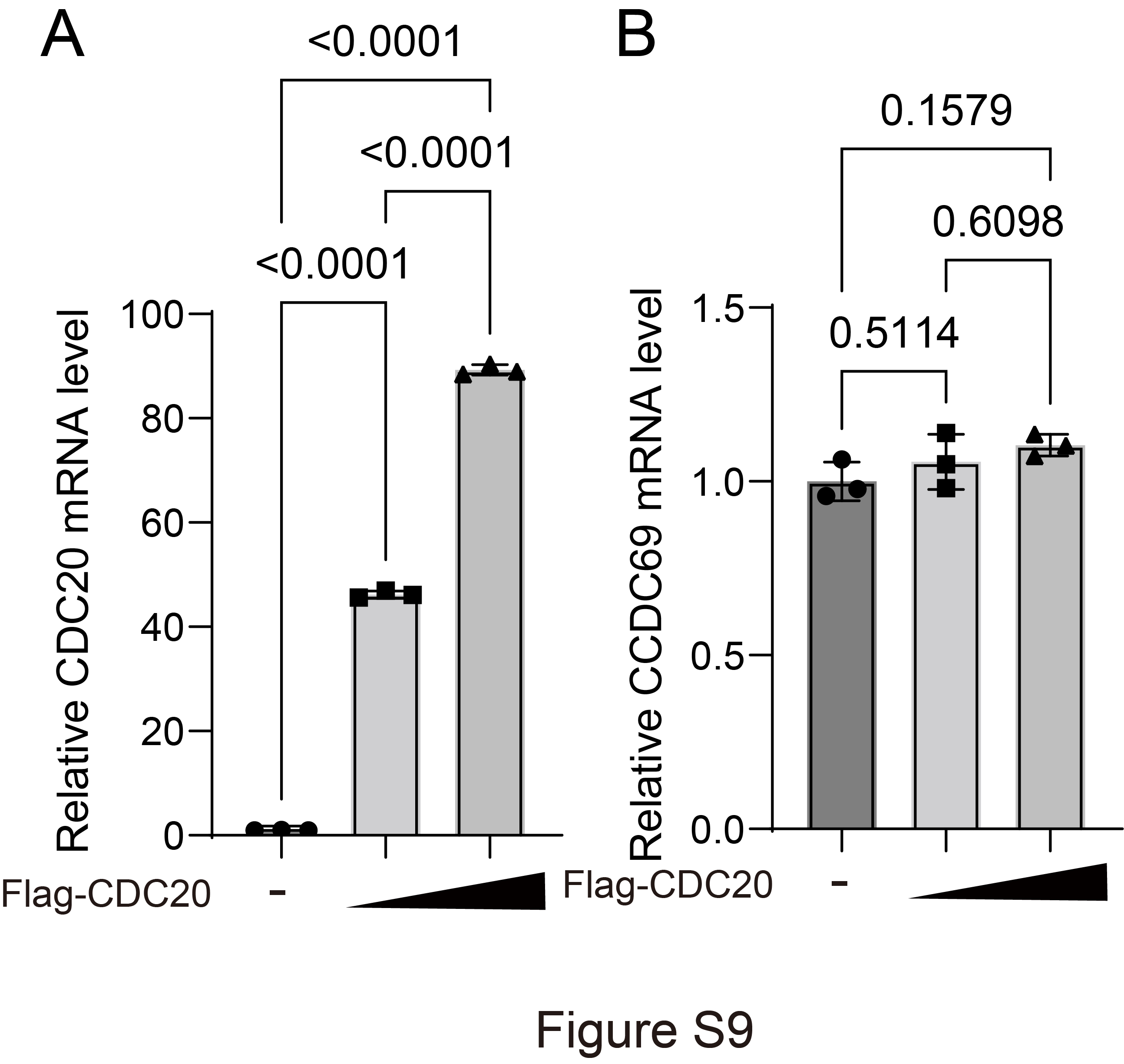


Fig. S9 Overexpression of CDC20 does not affect the mRNA expression level of CCDC69. (a, b) The mRNA expression levels of CDC20 and CCDC69 were detected by qPCR after transfecting CDC20 plasmids at different concentrations into 293T cells (n=3). qPCR: quantitative polymerase chain reaction. Data are represented as the mean ± SD. Statistical analysis was performed with one-way ANOVA.


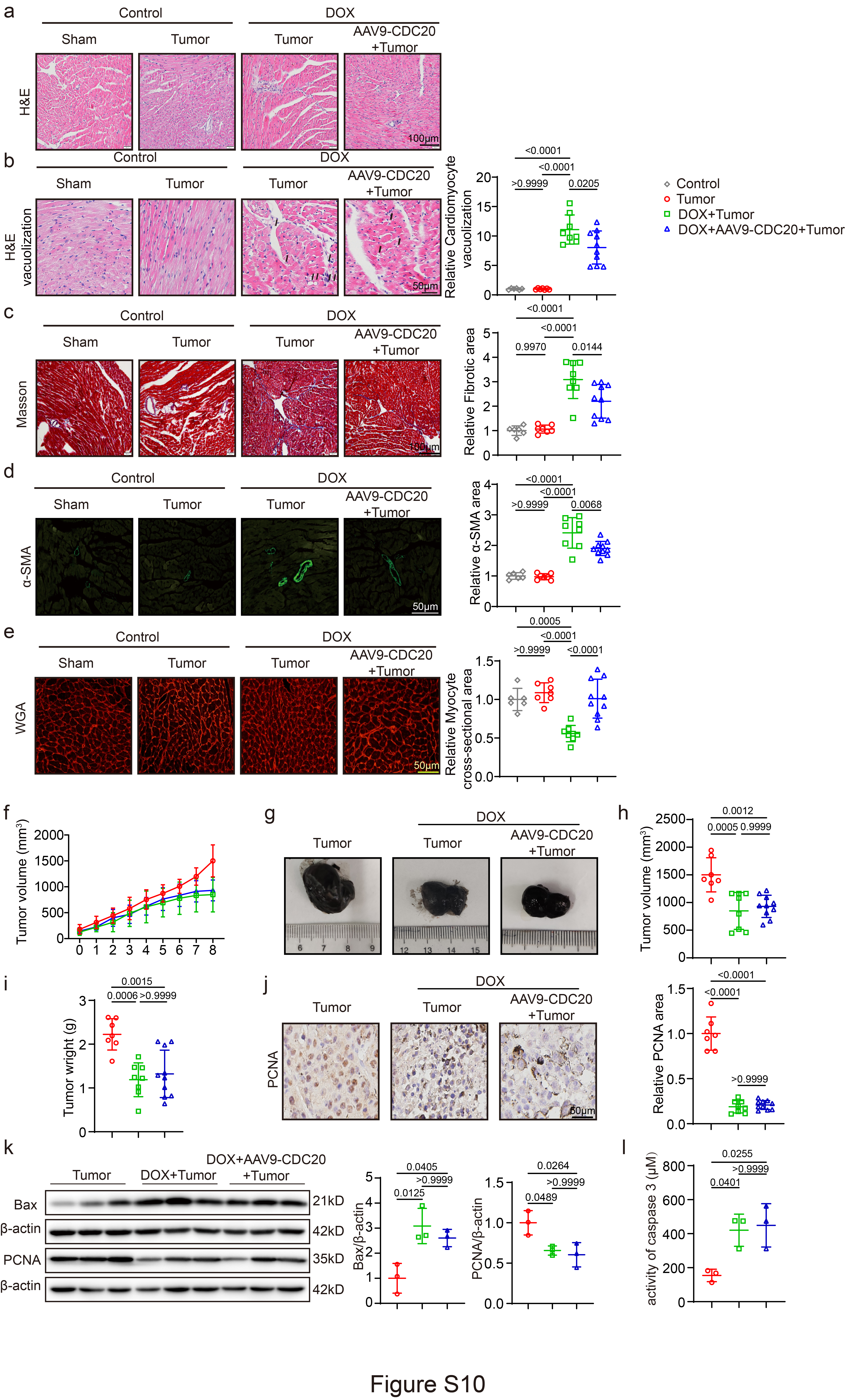


Fig.S10 Cardiomyocyte-specific overexpression of CDC20 significantly inhibits DOX-induced myocardial injury, while not affecting the antitumor effect of DOX. (a) Representative images of HE staining in each group (n=6-10, bar=100μm); (b) Representative images and statistical data of vacuolization in each group (n=6-10, bar=50μm); (c) Representative images and statistical data of Masson staining in each group (n=6-10, bar=100μm); (d) Representative images and statistical data of α-SMA staining in each group (n=6-10, bar=50μm); (e) Representative images and statistical data of WGA staining in each group (n=6-10, bar=50μm); (f) Tumor growth curves of mice in each group (n=7-10); (g) Representative images of tumor in each group; (h) Statistical data of tumor volume (n=7-10); (i) Statistical data of tumor weight (n=7-10); (j) Representative images and statistical data of PCNA staining in each group (n=7-10, bar=50μm); (k) Bax, PCNA and β-actin expression through western blot (n=3); (l) Caspase-3 activity in each group (n=3). α-SMA: alpha-smooth muscle actin, H&E: Hematoxylin and Eosin, HW/TL: heart weight/tibia length, PCNA: Proliferating Cell Nuclear Antigen, TUNEL: terminal deoxynucleotidyl transferase dUTP nick end labelling, WGA: wheat germ agglutinin. Data are represented as the mean ± SD. Statistical analysis was performed with two-way ANOVA.
